# Effects of prenatal exposure to (es)citalopram and maternal depression during pregnancy on DNA methylation and child neurodevelopment

**DOI:** 10.1038/s41398-023-02441-2

**Published:** 2023-05-05

**Authors:** Emilie Willoch Olstad, Hedvig Marie Egeland Nordeng, Geir Kjetil Sandve, Robert Lyle, Kristina Gervin

**Affiliations:** 1 Pharmacoepidemiology and Drug Safety Research Group, Department of Pharmacy, Faculty of Mathematics and Natural Sciences, University of Oslo, Oslo, Norway; 2 PharmaTox Strategic Research Initiative, Faculty of Mathematics and Natural Sciences, University of Oslo, Oslo, Norway; 3 UiO:RealArt Convergence Environment, University of Oslo, Oslo, Norway; 4 Department of Child Health and Development, Norwegian Institute of Public Health, Oslo, Norway; 5 Department of Informatics, Faculty of Mathematics and Natural Sciences, University of Oslo, Oslo, Norway; 6 Department of Medical Genetics, Oslo University Hospital and University of Oslo, Oslo, Norway; 7 Centre for Fertility and Health, Norwegian Institute of Public Health, Oslo, Norway; 8 Department of Research and Innovation, Division of Clinical Neuroscience, Oslo University Hospital, Oslo, Norway

**Keywords:** Biomarkers, ADHD, Epigenetics and behaviour, Predictive markers, Prognostic markers

## Abstract

Studies assessing associations between prenatal exposure to antidepressants, maternal depression, and offspring DNA methylation (DNAm) have been inconsistent. Here, we investigated whether prenatal exposure to citalopram or escitalopram ((es)citalopram) and maternal depression is associated with differences in DNAm. Then, we examined if there is an interaction effect of (es)citalopram exposure and DNAm on offspring neurodevelopmental outcomes. Finally, we investigated whether DNAm at birth correlates with neurodevelopmental trajectories in childhood. We analyzed DNAm in cord blood from the Norwegian Mother, Father and Child Cohort Study (MoBa) biobank. MoBa contains questionnaire data on maternal (es)citalopram use and depression during pregnancy and information about child neurodevelopmental outcomes assessed by internationally recognized psychometric tests. In addition, we retrieved ADHD diagnoses from the Norwegian Patient Registry and information on pregnancies from the Medical Birth Registry of Norway. In total, 958 newborn cord blood samples were divided into three groups: (1) prenatal (es)citalopram exposed (*n* = 306), (2) prenatal maternal depression exposed (*n* = 308), and (3) propensity score-selected controls (*n* = 344). Among children exposed to (es)citalopram, there were more ADHD diagnoses and symptoms and delayed communication and psychomotor development. We did not identify differential DNAm associated with (es)citalopram or depression, nor any interaction effects on neurodevelopmental outcomes throughout childhood. Trajectory modeling identified subgroups of children following similar developmental patterns. Some of these subgroups were enriched for children exposed to maternal depression, and some subgroups were associated with differences in DNAm at birth. Interestingly, several of the differentially methylated genes are involved in neuronal processes and development. These results suggest DNAm as a potential predictive molecular marker of later abnormal neurodevelopmental outcomes, but we cannot conclude whether DNAm links prenatal (es)citalopram exposure or maternal depression with child neurodevelopmental outcomes.

## Introduction

More than one in 10 women experience perinatal depression [1], and lasting depressive symptoms during pregnancy may contribute to both adverse maternal and child outcomes [2, 3]. To treat moderate to severe depression, pregnant women are increasingly prescribed antidepressants [4–6], with 1–7% of pregnant women using selective serotonin reuptake inhibitors (SSRIs) [4, 5, 7–9]. The structurally similar citalopram and escitalopram (hereafter, (es)citalopram) are collectively the most frequently prescribed SSRIs to pregnant women [4, 5, 8]. Pharmacoepidemiological studies have linked prenatal antidepressant exposure and maternal depression during pregnancy to an increased risk of abnormal neurodevelopmental outcomes in the child [10–12]. The underlying mechanisms are not known, but it has been shown that prenatal antidepressant exposure is associated with epigenetic differences in cord blood (in particular, DNA methylation [DNAm] of cytosine-phosphate-guanine sites [CpGs]) [13–15]. However, studies show conflicting results and are based on small sample sizes, candidate genes, and broad exposure definitions, and some lack a depression group to control for indication [14]. In five epigenome-wide association studies (EWASs) on prenatal antidepressant exposure and newborn cord blood DNAm, none of the differentially methylated CpGs overlap between any of the studies [15–19].

Studies have also investigated associations between prenatal exposure to antidepressants, DNAm in candidate genes, and child outcomes related to the central nervous system without significant findings [20–22]. While these studies are limited to a few candidate genes and investigate short-term outcomes, larger EWASs of long-term neurodevelopmental outcomes are needed. Associations between poor maternal mental health during pregnancy and DNAm differences in the offspring have also been shown, with several CpGs relevant to child neurodevelopment [23, 24]. Therefore, it is equally important to deconvolve the effect of prenatal exposure to antidepressants and unmedicated maternal depression on DNAm and altered neurodevelopment in the offspring.

Children with certain neurodevelopmental outcomes, such as attention-deficit/hyperactivity disorder (ADHD), show heterogeneity related to both phenotypic presentation and developmental course [25]. Interestingly, prospective studies have shown that DNAm measured at birth before symptom onset is associated with different ADHD symptom trajectories [26, 27]. Such results lend epigenetic insights into neurodevelopmental trajectories in childhood. However, whether prenatal environmental factors like prenatal antidepressant exposure and maternal depression may influence DNAm patterns associated with neurodevelopmental trajectories is not known.

In the present study, we have conducted epigenome-wide association analyses and investigated (1) whether prenatal exposure to (es)citalopram or maternal depression is associated with differences in DNAm in newborn cord blood, (2) the interaction effects of (es)citalopram and DNAm on long-term neurodevelopmental outcomes in the child, and (3) whether DNAm at birth is associated with later neurodevelopmental trajectories. This enabled a systematic investigation of the different aspects previously linked to the neurotoxicity of antidepressants by integrating maternal unmedicated depression and child neurodevelopmental outcomes in our EWAS.

## Methods

### Study population

This study is based on data and cord blood samples from the Norwegian Mother, Father and Child Cohort Study (MoBa) conducted by the Norwegian Institute of Public Health (NIPH) [28]. MoBa is an ongoing prospective, population-based birth cohort study (*n* = 114,500 children, *n* = 95,200 mothers, and *n* = 75,200 fathers), and 40.6% of women giving birth in Norway between 1998 and 2008 consented to participate. Participants complete questionnaires throughout pregnancy and in childhood. Cord blood samples were retrieved from the MoBa biobank, which contains blood samples from both parents during pregnancy, and from mothers and children (umbilical cord) at birth [29]. This study is based on data version 12 released by MoBa in 2020. MoBa was also linked to the Norwegian Patient Registry (NPR) and the Medical Birth Registry of Norway (MBRN).

The establishment of MoBa and initial data collection was based on a license from the Norwegian Data Protection Agency and approval from the Regional Committees for Medical and Health Research Ethics (REC). The MoBa cohort is currently regulated by the Norwegian Health Registry Act. All data were de-identified, and the linking of MoBa to health registries was handled by NIPH and the respective registries. Our study was approved by the REC South East Norway (reference: 23136, 2014/163).

### Sample selection and study design

Samples were selected specifically for this study into three groups: (1) prenatally (es)citalopram exposed, (2) prenatally maternal depression exposed, and (3) propensity score-selected controls (unexposed to antidepressants and maternal depression). The selection was based on MoBa questionnaires Q1 (gestational weeks 0–13), Q3 (weeks 13–29), and Q4 (week 30 to 6 months after delivery; selection into the present study included week 30 until birth). Only live, singleton births with cord blood samples available in the MoBa biobank were included. Women using antiepileptics and psycholeptics were excluded due to the potential teratogenic effects of these medications [30–37].

In the (es)citalopram group, other antidepressants were allowed, except when used concomitantly with (es)citalopram on the same indication. The indications for (es)citalopram were depression, anxiety, and other mental health problems. The depression group included women reporting depression, anxiety, or other mental health problems and exhibiting a mean depression symptom score ≥2.0 on either the Hopkins Symptom Checklist (SCL)-5 or -8. All samples available based on these selection criteria were included in the (es)citalopram or depression groups. The control group included women with no self-reported mental health problems and mean SCL-5 and -8 scores of 1.0 (no depressive symptoms), replying to both Q1, Q3, and Q4. Of the 17,228 women fulfilling these criteria, the final control group was selected by propensity scores, i.e., controls with similar propensities for (es)citalopram exposure as the subjects in the (es)citalopram group were selected. *PwrEWAS* [38] estimated that 300 subjects per group are sufficient to have 80% power to detect differences of 0.02 between groups (details in Supplementary Methods).

### Exposures

#### Prenatal (es)citalopram exposure

Citalopram (Anatomical Therapeutic Chemical [ATC]: N06AB04) is a mixture of the two stereoisomers *R*-citalopram and *S*-citalopram, and escitalopram (ATC: N06AB10) contains only the *S*-citalopram stereoisomer. Information about the maternal use of (es)citalopram was retrieved from the questionnaires Q1, Q3, and Q4 for 4-week intervals (gestational weeks 0–4; 5–8; 9–12; 13–16; 17–20; 21–24; 25–28; 30–birth). Prenatal (es)citalopram exposure was defined as reported use at either of these time points (in the Supplementary Information, see self-report validity in the Supplementary Methods and the distribution of (es)citalopram use across trimesters in Supplementary Fig. 1).

#### Maternal depression

Depression was assessed by two measures. The first measure was based on self-reported depression and recorded as answering “Yes” to having depression (Q1, Q3), anxiety (Q1), other psychological problems (Q3), or mental health problems (Q4) during pregnancy. Second, for the depression and control groups, we also included selection criteria on mean depression symptom scores from short versions of the SCL (SCL-5 in Q1 and SCL-8 in Q3; Supplementary Methods) [39–41]. A mean SCL-5 score ≥2.0 is indicative of depression [42, 43].

### Outcomes

#### DNA methylation

DNAm levels were measured using the Infinium MethylationEPIC BeadChip at Life & Brain (www.lifeandbrain.com/en/). Samples were randomly allocated to plates and beadchips, and processed as described previously [44]. The quality of the DNAm data was examined in the quality control module of *RnBeads* [45, 46]. Probes and samples that could bias the normalization and down-stream analyses were removed, including probes with SNPs (*n* = 17,371), cross-reactive probes (*n* = 43,463) [47], and poor-performing probes and samples with a detection *p*-value >0.01 (*n* = 18,435 probes; *n* = 1 sample). Then, background correction was done using the exponential-truncated-normal (ENmix) out-of-band (oob) method [48], followed by beta-mixture quantile (BMIQ) normalization [49]. After normalization, non-CpG probes (*n* = 1033) and probes on the sex chromosomes (*n* = 16,941) were removed. Finally, if *RnBeads*-estimated and MBRN-registered newborn sex differed, the sample was removed (*n* = 4). The final filtered data included 769,652 probes and 958 samples.

#### Neurodevelopmental outcomes

**Fig. 1:**
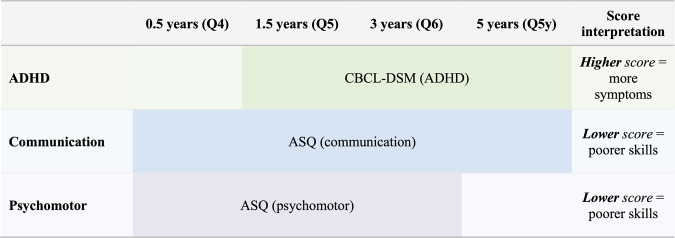
Overview of which questionnaires were used to assess neurodevelopmental outcomes in the children. ADHD attention-deficit/hyperactivity disorder, ASQ Ages and Stages Questionnaire, CBCL-DSM Child Behavior Checklist DSM-oriented subscale Q- MoBa questionnaire.

Child neurodevelopment was assessed using parental self-reports on internationally recognized psychometric tests at ages 0.5 years (Q4), 1.5 years (Q5), 3 years (Q6), and 5 years (Q5y). In addition, we retrieved ADHD diagnoses from the NPR recorded by specialists, registered as F90 in the 10th revision of the International Classification of Disease (ICD-10). The psychometric instruments included were the Child Behavior Checklist DSM-oriented (CBCL-DSM) ADHD subscale [50, 51] and the Ages and Stages Questionnaire (ASQ) communication and psychomotor subscales [52] (Fig. 1). These tests cover different domains of neurodevelopment. The psychomotor subscale covers both the fine and gross motor items of the ASQ. Age-of-onset of independent walking is an important milestone in gross motor development and, therefore, was also included in the analyses. In the CBCL-DSM, higher scores indicate more ADHD symptoms, and in the ASQ subscales, lower scores indicate possible developmental delays (Fig. 1; Supplementary Tables 1 and 2). Raw mean scores were standardized to *T* scores prior to statistical analysis (standardized to the entire MoBa population).

### Covariate assessment

We assessed potential covariates (listed in Supplementary Methods) in three steps. First, we performed principal component analysis (PCA) on the DNAm data and tested the associations between principal components (PCs) 1–3 and the covariates (one-way analysis of variance [categorical variables] and Spearman’s correlation test [continuous variables]; Supplementary Fig. 2A–B). Second, the individual contribution of the covariates significantly associated with DNAm variation was assessed by PC-PR^2^ (Supplementary Methods) [53, 54]. All covariates except bisulfite conversion and cell types contributed <1% of the DNAm variation (Supplementary Fig. 2C). Finally, we tested whether the covariates contributing the most to the DNAm variation differed between the comparison groups (Wilcoxon’s rank-sum test [continuous variables] and Chi-squared or Fisher’s exact test [categorical variables]; Supplementary Tables 3 and 4). Based on the results from these analyses, no covariates, other than those explicitly stated in the models below, were included.

Cell type composition (CD8^+^ and CD4^+^ T cells, natural killer cells, B cells, monocytes, granulocytes, and nucleated red blood cells [nRBCs]) was estimated using the *estimateCellCounts2* function implemented in *minfi* [55] and a validated cord blood reference (*FlowSorted.CordBloodCombined.450k*) [56, 57].

### Statistical analyses

#### Propensity scores

We generated the propensity scores using a logistic regression model to estimate the conditional probability of receiving (es)citalopram given defined pretreatment characteristics (prenatal paracetamol exposure, non-steroidal anti-inflammatory drugs [NSAIDs], opioid and antimigraine medication exposure, siblings, and maternal age, pre-pregnancy body mass index [BMI], education, income, lifetime history of major depression [LTHMD], smoking and alcohol consumption) [58, 59]. From these, we selected the covariates with a *p*-value <0.1 for inclusion in the final model matching the (es)citalopram subjects to controls: maternal income, BMI, LTHMD, smoking and alcohol at the start of pregnancy, and parity. We used nearest neighbor matching with a caliper width of 0.20 of the pooled standard deviation of the regression model (≈0.22) [59].

#### Trajectory analyses

Trajectory analyses of psychometric test scores over multiple time points were performed using latent class growth analysis (LCGA, also called group-based trajectory models), which is an unsupervised clustering method for longitudinal data [60]. We included the subjects from all three comparison groups in the analysis, having information at one time point or more about the relevant neurodevelopmental outcome. Models were run using the *lcmm* function in the *lcmm* R package [61], with maximum likelihood estimation (Supplementary Fig. 3). We examined 1–5 classes, using a linear or quadratic shape of time, and the *thresholds* link function, as suggested for psychometric test data [62]. Initial values were selected using an automatic grid search of 100 random value vectors. Each model was run for a maximum of 100 iterations; if a model did not converge, we increased to a maximum of 10,000 iterations. The final models were selected based on the goodness-of-fit and discriminatory power of the models, using the Akaike information criterion (AIC), the Bayesian information criterion (BIC), the sample size-corrected BIC (c-BIC) and entropy (Supplementary Tables 5–7). Lower AIC, BIC, and c-BIC indicate better relative model fit, while entropy close to 1 indicates good classification.

#### DNAm analyses

We used *β* values for visualization purposes and *M* values for statistical analyses [63]. To examine the three main objectives of this study, different models were run (see a detailed outline in Supplementary Fig. 4).

Investigation of the association of prenatal exposure to (es)citalopram or maternal depression with DNAm was performed by pairwise group comparisons performed by fitting linear regression models to mean DNAm in *limma* [64], defined by:

$${\mathrm{DNAm}} \sim \beta_{1} \,\, {\,}^\ast\,\, {\mathrm{group}}\, +\, \varepsilon.$$

The interaction was assessed by running logistic (ADHD diagnosis) or ordinal logistic regression models (CBCL-DSM and ASQ *T* scores at single time points and age-of-onset of walking):

$$\begin{array}{l}{\mathrm{Neurodevelopmental}}\,{\mathrm{outcome}} \,\sim\, \beta_{0} \,+\, \beta_{1}\,\, {\,}^\ast\,\, {\mathrm{(es)citalopram}}\\ +\, \beta_{2} \,\, {\,}^\ast\,\, {\mathrm{DNAm}}\,+\, \beta_{3} \,\, {\,}^\ast\,\, {\mathrm{(es)citalopram}} \,\, {\,}^\ast\,\, {\mathrm{DNAm}}\,+\, \varepsilon,\end{array}$$

where *β*_2_ represents the marginal effects of DNAm on the neurodevelopmental outcomes, and *β*_3_ represents the interaction between DNAm and (es)citalopram exposure. To identify the stable marginal effect of (es)citalopram exposure on neurodevelopmental outcomes, we reduced the model to run the model only once (Neurodevelopmental outcome ∼ *β*_1_ * (es)citalopram + *ε*). Ordinal logistic regression was used due to the highly skewed distributions of the *T* scores for some of the neurodevelopmental outcomes (Supplementary Fig. 5). To assess the effect of (es)citalopram and limit the impact of depression, the interaction models were run, including the (es)citalopram and depression groups only.

Finally, trajectory classes and DNAm associations were assessed by pairwise comparisons of trajectory classes in linear regression models:

$${\mathrm{DNAm}} \sim \beta_{0}\, +\, \beta_{1} \,\, {\,}^\ast\,\, {\mathrm{trajectory}}\,{\mathrm{class}}\,+\, \varepsilon.$$

All comparisons were adjusted for multiple testing with a false discovery rate (FDR) < 0.05, using the Benjamini and Hochberg method [65]. The test statistics of the EWAS on prenatal (es)citalopram and depression exposure on DNAm, were corrected for bias and inflation using the Bayesian method implemented in the R package *BACON* [66].

#### Analyses of significant CpGs

The annotation of CpGs was performed using the *IlluminaHumanMethylationEPICanno.ilm10b4.hg19* package [67]. The BECon web application [68] was used to assess the blood–brain correlation of the significant CpGs.

## Results

### Prenatal exposure to (es)citalopram and maternal depression, and DNA methylation patterns in cord blood

**Fig. 2:**
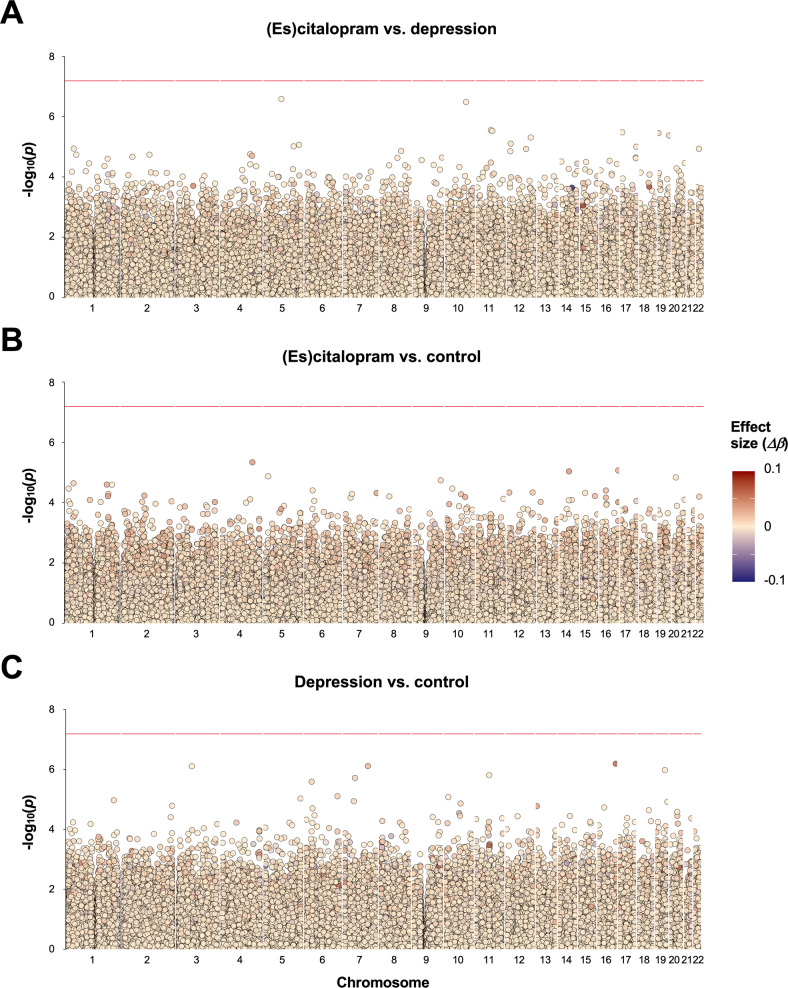
Modified Manhattan plots of difference in DNAm between groups. Log_10_ *p*-value against the genomic positions of the CpGs, after test statistic correction for bias and inflation using *BACON* [66]. Each dot represents a CpG, colored according to the DNAm difference between **A** (es)citalopram and depression groups, **B** (es)citalopram and control groups, and **C** depression and control groups. The red lines indicate the FDR-adjusted significance cutoff (<0.05).

We selected samples into three groups: (1) prenatally (es)citalopram exposed (*n* = 306), (2) prenatally maternal depression exposed (*n* = 308), and (3) propensity score-selected controls (*n* = 344). Sample characteristics are presented in Table 1. First, we ran PCA to identify potential covariates associated with variation in DNAm (Supplementary Fig. 2). This analysis revealed an association of the estimated nRBC proportion with DNAm variation, which contributed >5% of the variation explained by PCs 1–3 and was significantly different between the groups (Supplementary Fig. 2, Fig. 1 and Table 1). However, as the difference in mean nRBC proportion between groups was negligible (0.01–0.02), it was not included as a covariate in our models. Consequently, no covariates other than those stated below were included in the models. Then, we performed pairwise epigenome-wide association analyses between the groups to identify differential DNAm associated with prenatal exposure to (es)citalopram and maternal depression. These analyses did not reveal any significant differences in DNAm associated with prenatal (es)citalopram exposure or maternal depression (Fig. 2 and Supplementary Fig. 6).

**Table 1:** Overview of the comparison group characteristics.

	(Es)citalopram group (*n* = 306)	Depression group (*n* = 308)	Control group (*n* = 344)	*p*
**Maternal characteristics**				
** Maternal age** (mean years ± SD)	30.3 ± 5.2	28.4 ± 5.3	30.9 ± 4.6	^**a,c**^
** Pre-pregnancy BMI** (mean BMI ± SD)	24.5 ± 5.1	24.3 ± 4.8	23.8 ± 4.2	**N.S**.
	*8 NA*	*7 NA*	*2 NA*	
** Maternal education**				^**a,b,c**^
University/college (*n* (%))	178 (58.2)	136 (44.2)	247 (71.8)	
High school or lower (*n* (%))	124 (40.5)	163 (52.9)	89 (25.9)	
	*4 NA*	*9 NA*	*8 NA*	
** Smoking in pregnancy** (yes; *n* (%))	43 (14.1)	52 (16.9)	28 (8.1)	^**b,c**^
	*2 NA*			
** Alcohol in pregnancy** (yes; *n* (%))	36 (11.8)	28 (9.1)	64 (18.6)	^**c**^
	*49 NA*	*45 NA*	*15 NA*	
** Folic acid in pregnancy** (yes; *n* (%))	182 (59.5)	168 (54.6)	205 (59.6)	**N.S**.
**Maternal medications**				
** Analgesics** (yes; *n* (%))	190 (62.1)	191 (62.0)	178 (51.7)	^**b,c**^
** Antidepressants except (es)citalopram** (yes; *n* (%))	19 (6.2)	---	---	**---**
** NSAIDs** (yes; *n* (%))	55 (18.0)	45 (14.6)	30 (8.7)	^**b,c**^
**Maternal morbidities**				
Comorbidity index* (mean score ± SD)	0.5 ± 0.9	0.5 ± 0.9	0.4 ± 0.9	**N.S**.
	*27 NA*	*6 NA*	*13 NA*	
Chronic diseases**				**N.S**.
None (*n* (%))	277 (90.5)	280 (90.9)	325 (94.5)	
1–2 diseases (*n* (%))	27 (8.8)	28 (9.1)	19 (5.5)	
≥3 diseases (*n* (%))	0 (0)	0 (0)	0 (0)	
	*2 NA*			
** SCL-5** (mean score ± SD)	1.9 ± 0.8	2.8 ± 0.5	1.0 ± 0	^**a,b,c**^
	*16 NA*			
** SCL-8** (mean score ± SD)	1.7 ± 0.6	2.7 ± 0.5	1.0 ± 0	^**a,b,c**^
	*44 NA*			
** Lifetime history of major depression** (yes; *n* (%))	136 (44.4)	101 (32.8)	114 (33.1)	^**a,b**^
	*7 NA*	*9 NA*	*1 NA*	
**Child characteristics**				
** Birth weight** (mean grams ± SD)	3568 ± 501	3579 ± 512	3629 ± 503	^**b**^
			*1 NA*	
** Gestational age** (mean weeks ± SD)	39.4 ± 1.5	39.4 ± 1.6	39.7 ± 1.5	^**b,c**^
	*1 NA*	*1 NA*	*1 NA*	
** Infant sex** (female; *n* (%))	148 (48.4)	149 (48.4)	181 (52.6)	**N.S**.

*ADHD* attention-deficit/hyperactivity disorder, *ASQ* Ages and Stages Questionnaire, *BMI* body mass index, *CBCL-DSM* Child Behavior Checklist DSM-oriented subscale, *NA* missing value, *N.S*. not significant, *SCL* Hopkins Symptom Checklist, *SD* standard deviation.

*All variables available in MBRN and MoBa from the list by Bateman et al. [68, 69].

**Chronic diseases include asthma, rheumatoid arthritis, epilepsy, Crohn’s disease, lupus, multiple sclerosis (MS), cancer, and diabetes mellitus.

**Significant difference between:** ^a^the (es)citalopram and depression groups; ^b^the (es)citalopram and control groups; ^c^the depression and control groups.

### More ADHD symptoms and delayed communication and psychomotor skills among children exposed to (es)citalopram during pregnancy

Studies have reported an association between prenatal antidepressant exposure and child ADHD diagnosis, but results have been conflicting [10–12]. We observed a significantly higher proportion of children with ADHD in this study (*n* = 51, 5.3%) compared to the whole MoBa cohort (*n* = 3014, 3.0%; Fisher’s exact test, *p* < 0.00001). This was also evident when comparing the three sample groups in our study, where children prenatally exposed to (es)citalopram (7.5%) were significantly more likely to have an ADHD diagnosis compared to the controls (2.9%; Table 2). Children prenatally exposed to maternal depression also exhibited a higher proportion of ADHD diagnoses (5.8%) than the control group, but this difference was not significant. There were also significant differences between the comparison groups for several parent-reported neurodevelopmental outcomes (Table 2). ADHD symptoms were assessed using the CBCL-DSM, and communication and psychomotor skills were measured with the ASQ and age-of-onset of walking, assessed at 0.5 years, 1.5 years, 3 years, and 5 years. The raw mean scores of the questionnaires were standardized to *T* scores based on the entire MoBa population. In the CBCL-DSM, higher *T* scores indicate more ADHD symptoms, and in the ASQ, lower *T* scores indicate possible developmental delays (Fig. 1).

**Table 2:** Overview of child neurodevelopmental outcomes by comparison group.

	(Es)citalopram group (*n* = 306)	Depression group (*n* = 308)	Control group (*n* = 344)	*p*
**ADHD diagnosis**				
** ADHD diagnosis** (*n* (%))	23 (7.5)	18 (5.8)	10 (2.9)	^**b**^
**ADHD assessment**				
** CBCL-DSM (ADHD subscale)—1.5 years** (median *T* score ± SD)	46.3 (12.3)	52.5 (12.3)	46.3 (12.3)	^**a,c**^
	*94 NA*	*106 NA*	*58 NA*	
** CBCL-DSM (ADHD subscale)—3 years** (median *T* score ± SD)	52.1 (17.4)	52.1 (17.4)	47.7 (13.1)	^**b,c**^
	*140 NA*	*148 NA*	*103 NA*	
** CBCL-DSM (ADHD subscale)—5 years** (median *T* score ± SD)	51.7 (13.4)	56.2 (17.8)	47.3 (17.8)	^**a,b,c**^
	*178 NA*	*206 NA*	*177 NA*	
**Communication assessment**				
** ASQ (communication subscale)—0.5 years** (median *T* score (IQR))	55.3 (0)	55.3 (14.5)	55.3 (14.5)	**N.S**.
	*47 NA*	*50 NA*	*12 NA*	
** ASQ (communication subscale)—1.5 years** (median *T* score (IQR))	51.6 (19.7)	58.1 (13.1)	58.1 (13.1)	^**b**^
	*90 NA*	*103 NA*	*54 NA*	
** ASQ (communication subscale)—3 years** (median *T* score (IQR))	55.7 (8.5)	55.7 (8.5)	55.7 (8.5)	**N.S**.
	*141 NA*	*146 NA*	*106 NA*	
** ASQ (communication subscale)—5 years** (median *T* score (IQR))	55.8 (7.3)	48.5 (14.6)	55.8 (7.3)	^**b,c**^
	*189 NA*	*215 NA*	*199 NA*	
**Motor assessments**				
** ASQ (psychomotor subscale)—0.5 years** (median *T* score (IQR))	55.6 (9.0)	55.6 (9.0)	55.6 (9.0)	^**b**^
	*47 NA*	*49 NA*	*12 NA*	
** ASQ (psychomotor subscale)—1.5 years** (median *T* score (IQR))	55.4 (7.2)	55.4 (14.5)	55.4 (7.2)	**N.S**.
	*92 NA*	*102 NA*	*62 NA*	
** ASQ (psychomotor subscale)—3 years** (median *T* score (IQR))	51.3 (14.9)	51.3 (14.9)	51.3 (14.9)	**N.S**.
	*142 NA*	*151 NA*	*108 NA*	
** Age-of-onset of independent walking** (median number of months (IQR))	13.0 (2.3)	12.6 (2.4)	12.6 (2.1)	**N.S**.
	*147 NA*	*161 NA*	*114 NA*	

*ADHD* attention-deficit/hyperactivity disorder, *ASQ* Ages and Stages Questionnaire, *CBCL-DSM* Child Behavior Checklist DSM-oriented subscale, *IQR* interquartile range, *NA* missing value, *N.S*. not significant, *SD* standard deviation.

**Significant difference between:** ^a^the (es)citalopram and depression groups; ^b^the (es)citalopram and control groups; ^c^the depression and control groups.

### Interaction effects of DNAm and prenatal exposure to (es)citalopram on neurodevelopmental outcomes

While some studies suggest that prenatal exposure to antidepressants is associated with abnormal neurodevelopmental outcomes such as ADHD [10–12], little is known about molecular mechanisms underlying such associations. We investigated the potential interaction of DNAm and prenatal (es)citalopram exposure on several neurodevelopmental outcomes. Specifically, we examined the marginal and interaction effects of (es)citalopram exposure and DNAm on neurodevelopmental outcomes by comparing the children exposed to (es)citalopram and depression only. The outcomes included ADHD diagnosis and ADHD symptoms (CBCL-DSM) and ASQ-measured communication and psychomotor skills at single time points.

The marginal effects of (es)citalopram exposure on neurodevelopmental outcomes were only significant for ADHD symptoms at 1.5 and 5 years of age. The odds of children prenatally exposed to (es)citalopram exhibiting more ADHD symptoms at 1.5 years of age was 0.40 times that of children prenatally exposed to unmedicated maternal depression (confidence interval [CI]: [0.28, 0.57]; *p* < 0.000001) and 0.56 times at 5 years of age (CI: [0.35, 0.88], *p* < 0.01). The direction of the effect was equal at 3 years of age, albeit not statistically significant (odds ratio ≈ 0.72; CI: [0.49,1.06]; *p* < 0.1). The marginal effects of 23 CpGs on ASQ-measured psychomotor skills were significant at 3 years of age, annotating to 17 different genes (Supplementary Table 8). Several of these genes are important in neurogenesis (*TLE1*) [69] and neuronal differentiation (*GABPA*) [70], early embryonic development (*GABPA*) [71], and cellular growth and development (*DYRK2*) [72]. Further, mutations in some of the genes have been associated with neurological phenotypes, such as Aicardi-Goutierès syndrome (*RNASEH2C*) [73], intellectual disabilities and epilepsy (*DNM1*) [74], and autism spectrum disorder (*ARRB2*) [75]. Notably, the analyses did not identify significant interaction effects of (es)citalopram exposure and DNAm on any of the neurodevelopmental outcomes.

### DNAm at birth and later neurodevelopmental trajectories

Children with abnormal neurodevelopmental outcomes often present heterogeneity in the developmental course, and studies have shown that DNAm is associated with different neurodevelopmental trajectories [26, 27]. Hence, we investigated whether DNAm measured in cord blood at birth before symptom onset was associated with later neurodevelopmental trajectories of ADHD symptoms and communication and psychomotor development. Trajectories were estimated over three or four time points from 0.5 to 5 years after birth, depending on the neurodevelopmental outcome (Supplementary Tables 5–7).

**Fig. 3:**
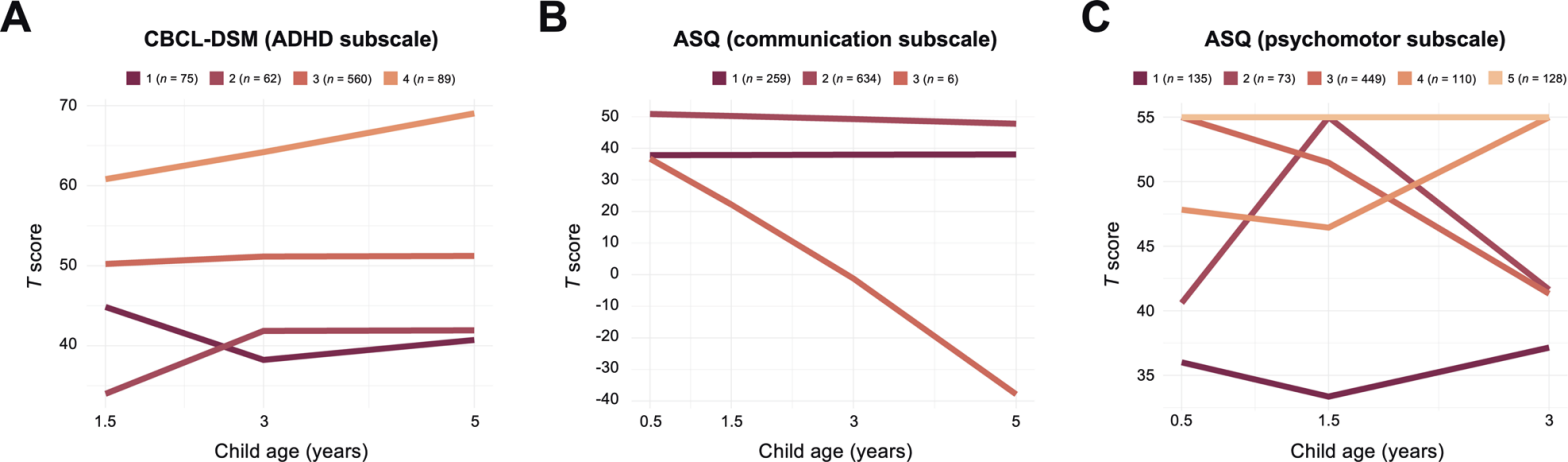
Neurodevelopmental trajectories identified using latent class growth analysis. Trajectories were identified for **A** the CBCL-DSM ADHD subscale, **B** the ASQ communication subscale, and **C** the ASQ psychomotor subscale.

Children were classified into trajectories following similar developmental patterns (Fig. 3). Specifically, trajectory analysis of the CBCL-DSM ADHD subscale classified children into four trajectories (Fig. 3A). Children in the two trajectories with the lowest CBCL-DSM *T* scores, indicating fewer ADHD symptoms (classes 1 and 2), showed similar developmental courses. A large proportion of children were classified into class 3, showing a moderate CBCL-DSM *T* score. Children in the highest trajectory (class 4) had a consistently high CBCL-DSM *T* score from 62 to 68 between 1.5 and 5 years of age, indicating more pronounced and slightly increasing ADHD symptomatology. As expected, the class 4 was significantly enriched with ADHD-diagnosed children and, notably, also exhibited a significantly higher proportion of children exposed to maternal depression (Supplementary Table 9).

The trajectory analyses of the ASQ communication and psychomotor subscales classified children into three and five trajectory classes, respectively (Fig. 3B–C, Supplementary Tables 10 and 11). Of note, the ASQ communication trajectory class 3 contained only six children following a very different developmental course compared to the other children (Fig. 3B). The children in the three study comparison groups were evenly distributed between the trajectory classes (Supplementary Tables 10–11). In conclusion, these results clearly demonstrate heterogeneity in the developmental course of the different outcome measures between children.

Next, to investigate whether DNAm at birth may be a potential biomarker of later developmental trajectories reflecting symptom severity, we performed epigenome-wide analyses and compared DNAm between the identified trajectories. For the CBCL-DSM ADHD subscale trajectories, children in the two classes showing the lowest *T* scores (class 1 and 2; Fig. 3) and, therefore, unlikely to have ADHD, were grouped together in the analyses. We pairwise compared the three trajectories and found no significant associations between cord blood DNAm at birth and the trajectories.

For the ASQ communication subscale trajectories, we excluded trajectory class 3 containing only six children, and compared DNAm between classes 1 and 2 (Fig. 3B). Multiple CpGs (*n* = 254) were differentially methylated between the two ASQ communication trajectories (Supplementary Table 8). Interestingly, two CpGs annotated to *PEX10*, involved in peroxisomal processes, have previously been identified in child saliva associated with ADHD [76], in cord blood associated with ADHD trajectories [26], and upon prenatal exposure to paracetamol in children with ADHD [44]. Also, four CpGs are annotated to the *BEGAIN* gene, which is involved in the regulation of postsynaptic neurotransmitter receptor activity in the brain. Other genes of interest are three CpGs located in *HOXC4*, which is involved in the development of the nervous system, one CpG in *KCNJ5* previously associated with ADHD [44], and one CpG in *SHANK2,* which is involved in transmission in excitatory neurons. Mutations in the *SHANK2* gene have been associated with both ADHD and autism spectrum disorder [77].

**Fig. 4:**
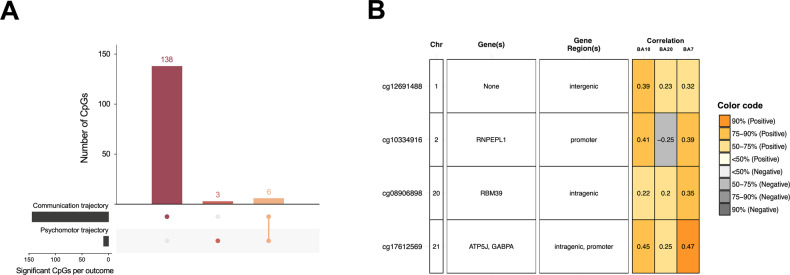
CpGs associated with developmental trajectories and blood–brain correlation of DNAm. **A** Upset plot [83, 84] shows the overlap of significant CpGs associated with communication and/or psychomotor developmental trajectories. Overlapping CpGs are indicated by filled dots for the respective outcomes. The vertical bar plot indicates the number of CpGs for the particular intersection. **B** Blood–brain correlation of significant CpGs associated with both communication and psychomotor trajectories. Correlation is reported as Spearman’s correlation coefficient of DNAm between blood and brain. The modified plot from the BECon web application [68].

For the ASQ psychomotor subscale trajectories, pairwise comparisons of DNAm between all five trajectory classes (Fig. 3C) revealed differentially methylated CpGs between trajectory classes 3 and 4 (*n* = 32 CpGs annotated to 24 genes). Interestingly, several of these overlapped with differentially methylated CpGs identified between the communication trajectories, annotated to the *RFTN1, ERV3-1*, *RBM39*, *SHANK2*, *DYRK2*, *GABPA*, *ATP5J*, *PEX10*, *FAM45A*, *FAM45B*, *RNASEH2C*, *PPP1R12B* and *PRKXP1* genes (*n* = 16 CpGs annotated to 13 genes; Fig. 4A). Notably, nine of the 23 CpGs with significant marginal effects on psychomotor skills at 3 years also overlapped with common trajectory CpGs, annotated to *RFTN1*, *ERV3-1*, *RBM39*, *DYRK2*, *GABPA*, *ATP5J*, *FAM45A*, *FAM45B*, *RNASEH2C*, and *PPP1R12B*. As described, several of these genes are implicated in development (e.g., *DYRK2* [72] and *TGFB* [78]), neuronal differentiation (*GABPA* [70]), and neurological phenotypes, including ADHD (*RNASEH2C* [73], *PEX10* [26, 44, 76], and *SHANK2* [77]).

### Blood–brain DNAm correlation

To strengthen the mechanistic insights and interpretation of the significant DNAm findings in cord blood, we used BECon [68] to look up the correlation of DNAm in blood and brain tissue for the CpGs associated with the communication or psychomotor trajectories, as well as the 23 CpGs associated with psychomotor skills at 3 years. Of these 23 CpGs, nine CpGs exhibited blood–brain correlation data, all of which exhibited relatively weak blood–brain correlations between −0.5 and 0.5. Data on blood–brain correlations were available for 145 of the 254 CpGs associated with the communication trajectories. Of these, most CpGs exhibited relatively weak correlations between −0.5 and 0.5 (*n* = 141 CpGs), while four CpGs were positively correlated (>0.5) between blood and brain (Supplementary Table 12). Of the 32 significant CpGs associated with the psychomotor trajectory classes 3 and 4, 14 had blood–brain correlation data available in BECon. Among these CpGs, 12 showed a weak correlation (−0.5 < *R* < 0.5), and two CpGs were positively correlated (Supplementary Table 12). In Fig. 4B, the five CpGs available in BECon of the 16 CpGs associated with both the communication and psychomotor trajectories are shown. A CpG annotated to the *PRKXP1* gene was positively correlated with overall brain DNAm at this CpG (Fig. 4B). Further, one of the CpGs annotated to the *PEX10* gene exhibited a positive correlation with one brain area (BA10; Fig. 4B). In summary, these findings suggest that several of the significant CpGs identified in our study likely reflect DNAm levels in the brain.

## Discussion

We performed epigenome-wide association analyses and investigated whether prenatal exposure to (es)citalopram or maternal depression was associated with differences in cord blood DNAm at birth. To explore the role of DNAm on child neurodevelopmental outcomes associated with prenatal (es)citalopram exposure, we investigated the interaction effect on neurodevelopment. We also examined whether DNAm at birth was associated with later developmental trajectories of ADHD symptoms and communication and psychomotor skills. To our knowledge, this is the largest EWAS to date deconvolving associations of DNAm, prenatal (es)citalopram exposure, and maternal depression and assessing the potential effects on long-term neurodevelopmental outcomes.

The initial EWAS on (es)citalopram and maternal depression did not identify any differentially methylated CpGs compared to controls. Consequently, we did not replicate previous findings showing the association between prenatal antidepressant exposure or maternal depression and DNAm [15–19, 79], and there are several possible explanations for this [13, 14]. For example, previous EWASs are based on small sample sizes, varying genome coverage, and heterogenous methodologies [13, 14].

In line with previous studies [10–12], we observed differences in the proportions of ADHD diagnoses across the study groups, specifically when comparing children prenatally exposed to (es)citalopram to controls. There was also an increased proportion of ADHD diagnoses in the depression group compared to controls, albeit not significant. In MoBa, there are several parent-reported psychometric tests of neurodevelopment, including CBCL and ASQ, assessed between 0.5 and 5 years of age. We chose the CBCL-DSM ADHD subscale to measure ADHD symptoms and symptom heterogeneity [25, 80] and to possibly identify children with subthreshold ADHD. The ASQ communication and psychomotor subscales were included as the ASQ is an internationally recognized and widely used psychometric test, and covers other domains of neurodevelopment which can be, but are not necessarily, related to ADHD [25]. There were significant differences in several of the psychometric test *T* scores between the different groups. Trajectory analyses classified children into developmental trajectories of the CBCL-DSM ADHD subscale and the ASQ subscales of communication and psychomotor skills. The trajectories of ADHD symptom development are similar to trajectories identified previously [26]. Taken together, our results emphasize the importance of taking symptom heterogeneity and developmental course into consideration when assessing neurodevelopment in the prenatal pharmacoepigenetic context.

Whether DNAm is important in the association of prenatal antidepressants or depression exposure with child abnormal neurodevelopment is not known. Identification of molecular biomarkers for early risk detection of ADHD and related neurodevelopmental outcomes could potentially aid in the identification of children in need of early intervention and support. In this respect, DNAm patterns in cord blood measured at birth before the manifestation of symptoms are potentially particularly useful. Trajectories of communication and psychomotor development were associated with differential cord blood DNAm of genes previously associated with ADHD trajectories in childhood [26, 76]. Multiple genes were also involved in cellular growth, development, and neurological phenotypes. Interestingly, several of the differentially methylated genes also overlapped between the communication and psychomotor trajectories, suggesting a common effect. Although communication and psychomotor trajectories are not specific to ADHD, the complex etiology underlying ADHD is often accompanied by learning problems and motor and/or speech delays [25]. Between the communication trajectories, we found differential DNAm of *PEX10*, which encodes a protein functioning in peroxisomal processes. Such processes have been implicated in fatty acid oxidation in ADHD and have also been reported by Walton et al. [26] and Wilmot et al. [76]. While only one CpG in *PEX10* appeared to positively correlate between blood and brain, our results nevertheless suggest DNAm at birth as a potential molecular biomarker of later neurodevelopmental trajectories in children prenatally exposed to (es)citalopram and depression.

Our study has several limitations and strengths. This study is, to our knowledge, the largest prospective EWAS on antidepressants and DNAm; it may still be underpowered to detect DNAm differences associated with (es)citalopram and maternal depression. In particular, interaction models may inherently decrease power, and the psychometric tests at higher ages exhibit a pronounced decrease in respondents, mostly due to loss to follow-up [81]. To partly circumvent this limitation, the LCGA handled missing data when the score for at least one time point was known using maximum likelihood estimation. The loss to follow-up seemed to be differentially distributed among the comparison groups, with more depressed women lost to follow-up. This may bias our results towards the null, as women with more depressive symptoms are missing. We attempted to limit confounding by indication by including a depression group. However, the depression group scored significantly higher on the SCL-5 and -8, suggesting more severe depression symptoms at the time of reporting, likely due to being unmedicated. Therefore, we cannot exclude residual confounding by the severity of depression, as well as other unmeasured confounders. Finally, there is a known genetic component of ADHD, which we could not assess in the present study, as genotype data from MoBa Genetics were not available when the study was conducted. Future studies, including integrated analyses of genetic information, would enable investigations of genetic susceptibility to ADHD. The main strengths of the present study include the relatively large sample size and a focus on one specific antidepressant, compared to other published EWASs on prenatal antidepressant exposure [14, 15, 79]. Moreover, we also used propensity scores to select the unexposed control group, i.e., controls with similar propensities for (es)citalopram exposure, as the subjects in the (es)citalopram group were selected, thereby improving the inference of causation [58]. Finally, we cover multiple different domains of neurodevelopment at several time points throughout early life and also assess ADHD at both the diagnosis and symptoms level [82].

In conclusion, we did not identify significant differences in DNAm associated with prenatal exposure to (es)citalopram or maternal depression. There were more ADHD symptoms, as well as delayed communication and psychomotor skills, among children exposed to (es)citalopram compared to the controls. Differences in DNAm were associated with child neurodevelopmental trajectory classes reflecting symptom severity. Consequently, CpGs’ DNAm may be potential predictive molecular markers of later abnormal neurodevelopmental outcomes. Future studies are needed for replication and assessment of a functional impact on neuronal differentiation and developmental processes in model systems. Additionally, it will also be important to improve causal inference by integrating genetic data and simulating causal relationships using machine learning approaches on real-world and artificial data. This can elucidate the properties of causal relationships in observational studies using molecular data.

## Supplementary information

Supplementary Information

Supplementary Information, Supplementary Table 8

Supplementary Information, Supplementary Table 12

## Data Availability

The data that support the findings of this study are available from the Norwegian Mother, Father and Child Cohort Study, but restrictions apply to the availability of these data and so are not publicly available. However, data are available from the authors upon reasonable request and with permission from the Norwegian Mother, Father and Child Cohort Study.
