# Supplementary material for: Effects of prenatal exposure to (es)citalopram and maternal depression during pregnancy on DNA methylation and child neurodevelopment
Source: Transl Psychiatry. 2023 May 5;13:149. doi: 10.1038/s41398-023-02441-2 (PMC10163054; doi:10.1038/s41398-023-02441-2)
Supplement: Supplementary file 1 — Supplementary Information [file 41398_2023_2441_MOESM1_ESM.docx]

**Supplementary Information**

**Supplementary Methods**

# **Power calculation**

Previous epigenetic epidemiology reviews [1, 2], and our recent systematic literature review of prenatal pharmacoepigenetic studies [3], propose that small effect sizes may be expected and changes may be as low as 2 percentage points. Based on this, we used *pwrEWAS* [4] to estimate the power for different total sample sizes and expected effect sizes (Supplementary Figure 7). The estimation also accounted for tissue type (cord blood), false discovery rate (FDR; 0.05), differential methylation method (*limma*), and expected number of significant CpGs (both 100, 1,000, 3,000, and 6,000 CpGs were tested, but more than 1,000 significant CpGs did not change the results considerably; data not shown).

The power analyses show that to detect effect sizes as small as 0.02 (difference in DNAm level), 300 subjects per group are sufficient, provided that we expect 100 significant CpGs (Supplementary Figure 7A). However, if the expected number of significant CpGs is 1,000, we will have approximately 70% power to detect an effect size of 0.02, with the same number of subjects (300 subjects per group; Supplementary Figure 7B).

# **Validity of exposure and outcome measures**

# *Prenatal (es)citalopram exposure*

We used maternal self-reports of (es)citalopram use in MoBa. MoBa antidepressant self-reports and redeemed prescriptions registered in the Norwegian Prescription Database (NorPD) have shown good agreement, with 87.0% of reports of antidepressant use in MoBa also found to have filled a prescription for the antidepressant [1].

# *Maternal depression and depressive symptoms*

The Hopkins Symptom Checklist is an internationally recognized instrument to measure symptoms of mental disorders, including depression and anxiety [2, 3]. The original instrument consists of 90 items. However, MoBa includes shorter versions of 5 or 8 items (SCL-5 and -8, respectively), relating specifically to symptoms of depression and anxiety [4]. SCL-5 and -8 have been shown to correlate well with the full-item instrument (0.90 and 0.92 respectively) [4]. The SCL-5 has a sensitivity 82.0% and a specificity of 96.0% [5].

# *Neurodevelopmental outcomes*

The Child Behavior Checklist DSM-oriented (CBCL-DSM) ADHD subscale, and the Ages and Stages Questionnaire (ASQ) communication and psychomotor (covering fine and gross motor) subscales were used to measure different domains of neurodevelopment. When compared to ADHD diagnoses from semi-structured clinical interviews, the CBCL-DSM ADHD subscale exhibits a moderate agreement (*κ* = 0.51), a sensitivity of 81% and a specificity of 70% [6]. Only a subset of the original ASQ items were included in MoBa and these items span different age ranges in the ASQ questionnaires, to introduce more variation in scores across individuals (Supplementary Table 2). Overall, the ASQ exhibits good agreement (84%) compared to standardized assessments, having a sensitivity of 72% and a specificity of 86% [7]. The Norwegian version of the ASQ has also been validated [8].

# **Covariate assessment**

When assessing covariates for inclusion in the regression models, we analyzed covariates previously included in prenatal pharmacoepigenetic studies [9]: maternal age and BMI, marital status, maternal education, alcohol use, smoking, multivitamin use, psychotropic and analgesic use, maternal morbidities, Caesarean section, gestational age, infant sex, birth weight, bisulfite conversion and the estimated composition of white blood cells. The PC-PR^2^ method is extensively described elsewhere [10, 11]. Briefly, for each of the PCs 1–3 we fit linear regression models on the covariates. We computed the partial R^2^ for each covariate, reflecting the variation contribution of individual covariates to the total variation in the DNAm PC, when accounting for the contribution of the other covariates.

**References**

1. Skurtveit S, Selmer R, Odsbu I, Handal M. Self-reported data on medicine use in the Norwegian Mother and Child cohort study compared to data from the Norwegian Prescription Database. Nor Epidemiol. 2014;24:209–16.
2. Derogatis LR, Lipman RS, Covi L. The SCL-90: an outpatient psychiatric rating scale. Psychopharmacol Bull. 1973;9:13–28.
3. Derogatis LR, Lipman RS, Rickels K, Uhlenhuth EH, Covi L. The Hopkins Symptom Checklist (HSCL): a self-report symptom inventory. Behav Sci. Behav Sci; 1974;19:1–15.
4. Tambs K, Røysamb E. Selection of questions to short-form versions of original psychometric instruments in MoBa. Nor Epidemiol. 2014;24:195–201.
5. Strand BH, Dalgard OS, Tambs K, Rognerud M. Measuring the mental health status of the Norwegian population: a comparison of the instruments SCL-25, SCL-10, SCL-5 and MHI-5 (SF-36). Nord J Psychiatry. 2003;57:113–8.
6. Skarphedinsson G, Jarbin H, Andersson M, Ivarsson T. Diagnostic efficiency and validity of the DSM-oriented Child Behavior Checklist and Youth Self-Report scales in a clinical sample of Swedish youth. PLoS One. 2021;16.
7. Squires Potter, L., & Bricker, D J. The ASQ User’s Guide. ASQ User’s Guid. Paul H. Brookes; 1999.
8. Richter J, Janson H. A validation study of the Norwegian version of the Ages and Stages Questionnaires. Acta Paediatr. 2007;96:748–52.
9. Olstad EW, Nordeng HME, Gervin K. Prenatal medication exposure and epigenetic outcomes: a systematic literature review and recommendations for prenatal pharmacoepigenetic studies. Epigenetics. 2021.
10. Fages A, Ferrari P, Monni S, Dossus L, Floegel A, Mode N, et al. Investigating sources of variability in metabolomic data in the EPIC study: the Principal Component Partial R-square (PC-PR2) method. Metabolomics. 2014;10:1074–83.
11. Perrier F, Novoloaca A, Ambatipudi S, Baglietto L, Ghantous A, Perduca V, et al. Identifying and correcting epigenetics measurements for systematic sources of variation. Clin Epigenetics. 2018;10.
12. Conway JR, Lex A, Gehlenborg N. UpSetR: an R package for the visualization of intersecting sets and their properties. Bioinformatics. 2017;33:2938–40.
13. Lex A, Gehlenborg N, Strobelt H, Vuillemot R, Pfister H. UpSet: Visualization of Intersecting Sets. IEEE Trans Vis Comput Graph. 2014;20:1983–92.
14. van Iterson M, van Zwet EW, Heijmans BT, Consortium the B. Controlling bias and inflation in epigenome- and transcriptome-wide association studies using the empirical null distribution. Genome Biol. 2017;18:19.
15. Achenbach TM, Ruffle TM. The Child Behavior Checklist and related forms for assessing behavioral/emotional problems and competencies. Pediatr Rev. 2000;21:265–71.
16. Bateman BT, Mhyre JM, Hernandez-Diaz S, Huybrechts KF, Fischer MA, Creanga AA, et al. Development of a comorbidity index for use in obstetric patients. Obstet Gynecol. 2013;122:957–65.
17. Easter SR, Bateman BT, Sweeney VH, Manganaro K, Lassey SC, Gagne JJ, et al. A comorbidity-based screening tool to predict severe maternal morbidity at the time of delivery. Am J Obstet Gynecol. 2019;221:271.e1-271.e10.

**Supplementary Figures**


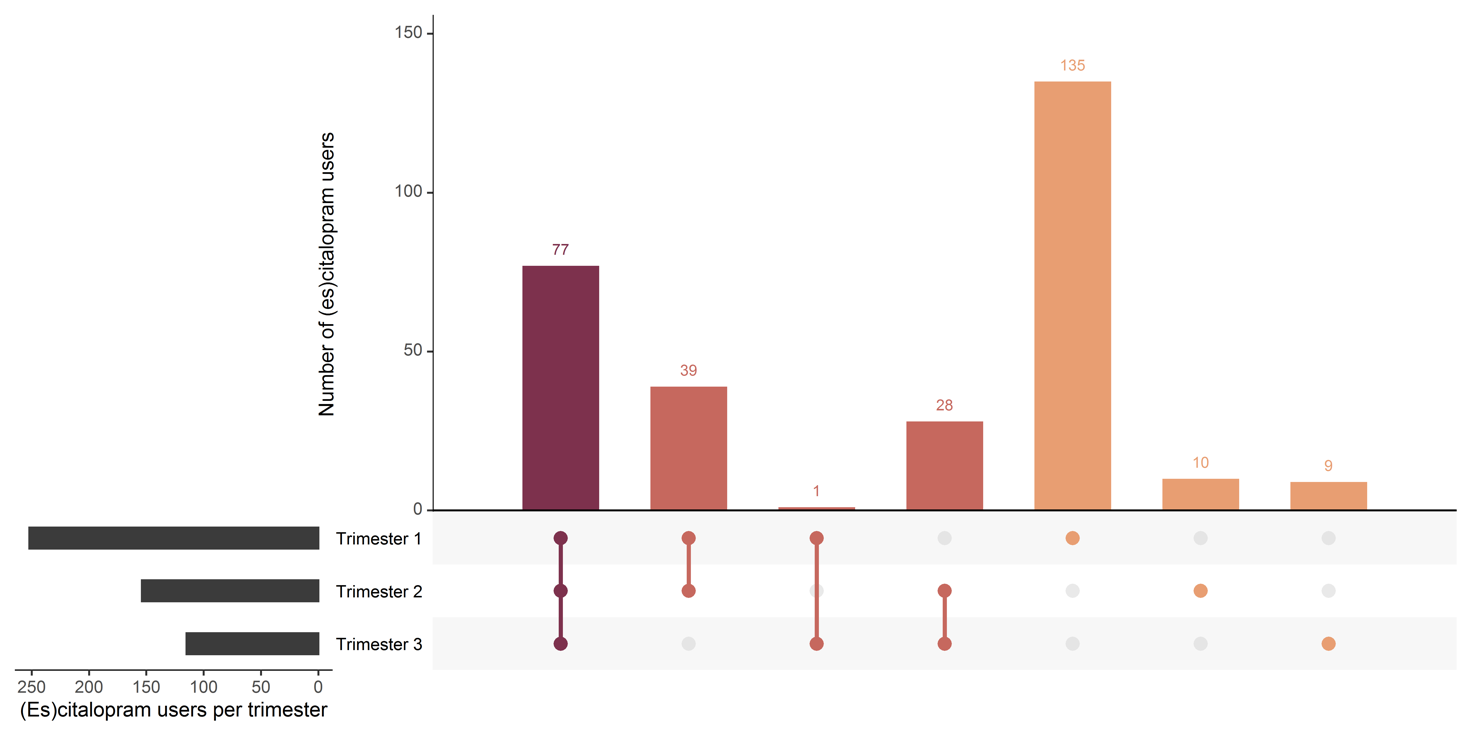


**Supplementary Figure 1. Distribution of maternally reported (es)citalopram use per trimester.**

UpSet plot [12, 13] showing the number of (es)citalopram users per trimester (horizontal, black bars), and the distribution of women reporting (es)citalopram use for either all three trimesters (purple), two trimesters (red) or one trimester only (orange).

**
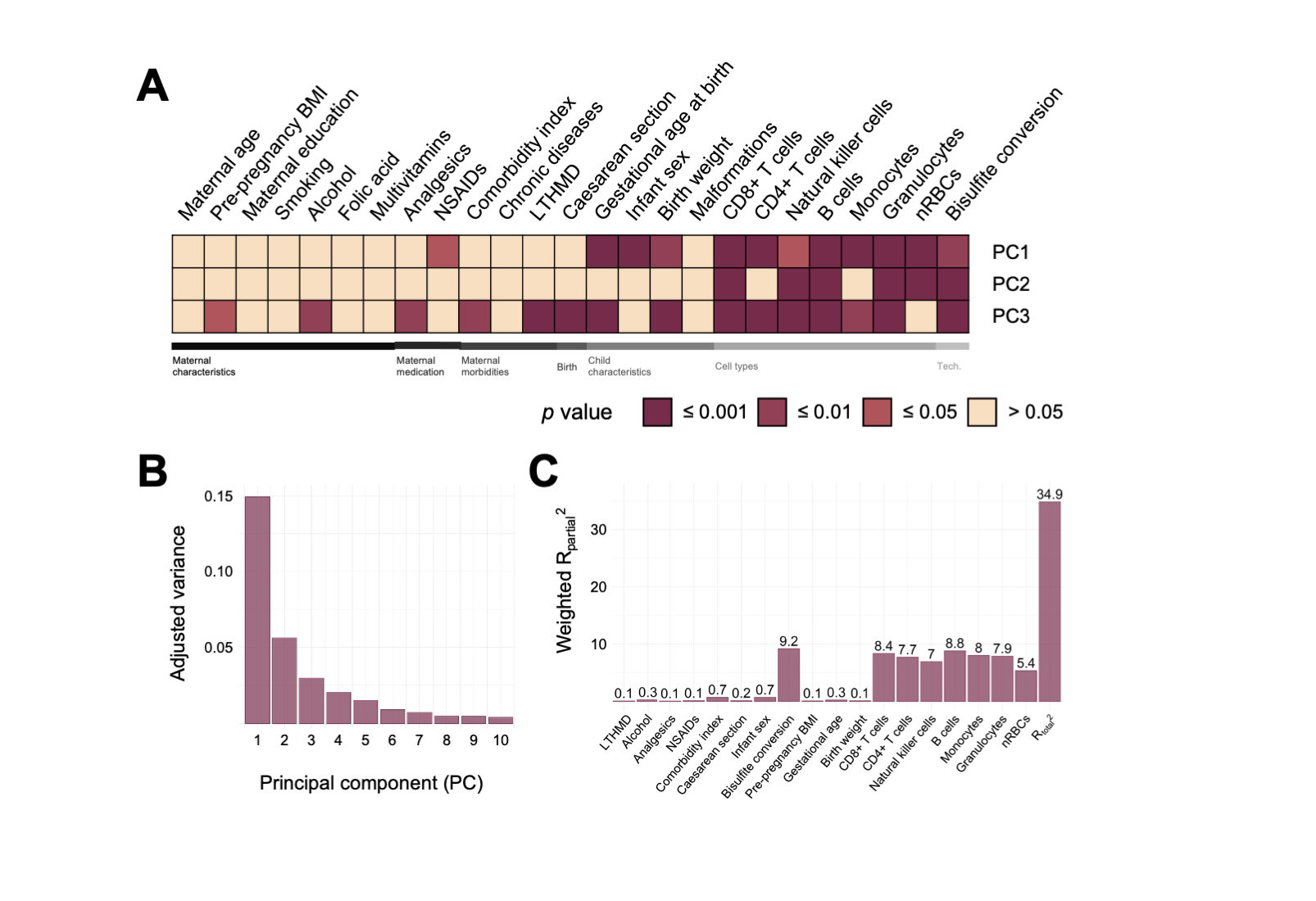
**

**Supplementary Figure 2. Principal component analysis (PCA) and PC-PR^2^ to investigate potential covariates.**

**(A)** Heat map of the association between different covariates and the top three principal components (PCs) representing the most DNAm variation. **(B)** Scree plot indicating the 10 first PCs’ contribution to DNAm variation. **(C)** Weighted partial R^2^ for each covariate significantly associated with either of the first 3 PCs (i.e., the variability contributed by the respective covariate to the top three PCs, when accounting for the variability contribution of all other covariates in the model). **Abbreviations:** BMI: body mass index; nRBC: nucleated red blood cell; LTHMD: life-time history of major depression; NSAID: non-steroidal anti-inflammatory drug; Tech.: technical covariate.


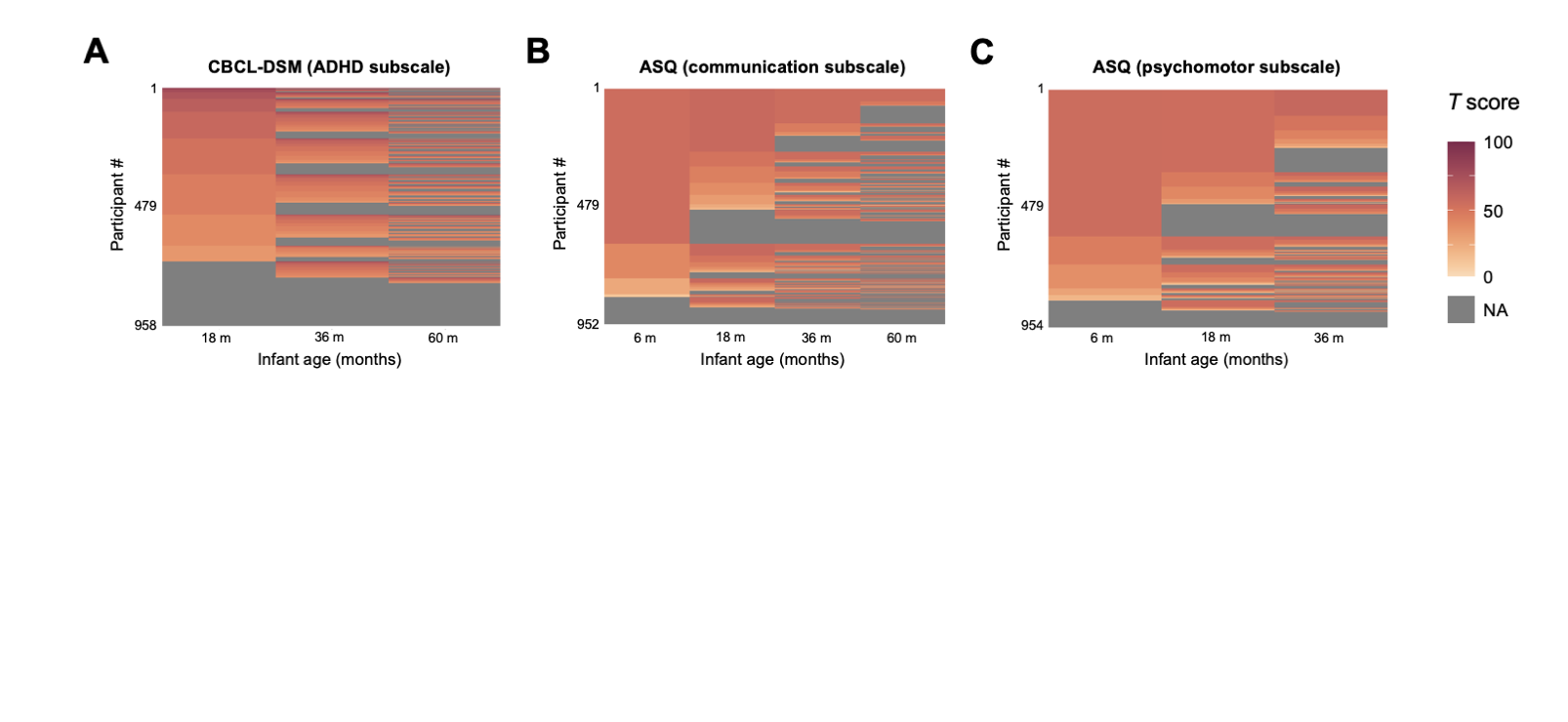


**Supplementary Figure 3. Lasagna plots showing the T score for every participant per time point reported.**

The development of the T score for each participant over the time points at which the respective psychometric test was distributed, for **(A)** CBCL-DSM (ADHD subscale), **(B)** ASQ (communication subscale), and **(C)** ASQ (psychomotor subscale). Subjects with T scores < 0 were removed for visualization purposes, but were included in statistical analyses (n_comm_ = 6 subjects and n_motor_ = 4 subjects). **Abbreviations:** ADHD: attention-deficit/hyperactivity disorder; ASQ: Ages and Stages Questionnaire; CBCL-DSM: Child Behavior Checklist, Diagnostic and Statistical Manual of Mental Disorders subscale.


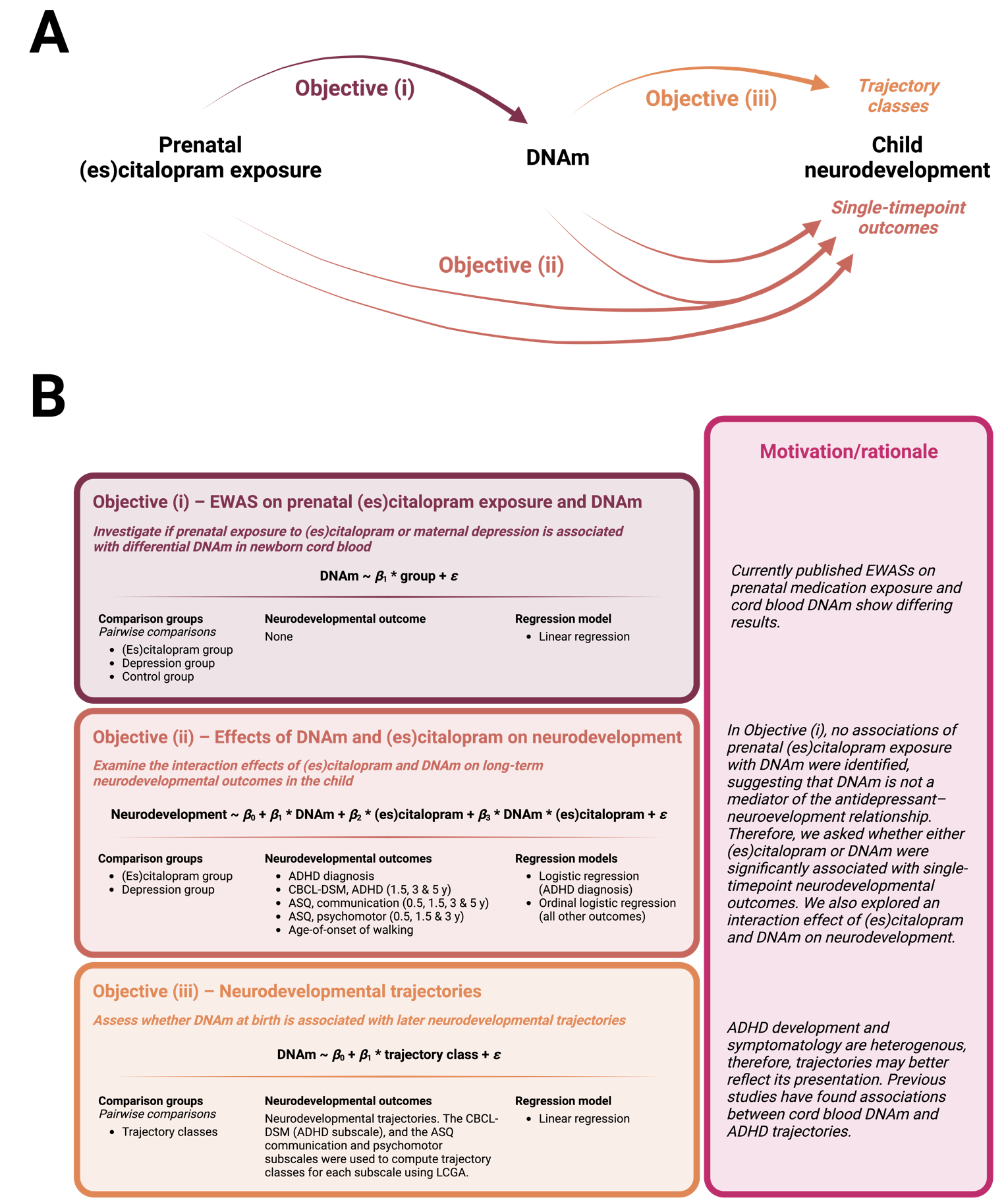


**Supplementary Figure 4. Overview of how each of the three objectives of the study were examined.**

**(A)** Schematic of which association(s) were tested for each objective. For Objective (ii) the lines indicate the marginal effects of (es)citalopram and DNAm on neurodevelopment (single-tailed arrows) and the interaction effects of (es)citalopram and DNAm on neurodevelopment (two-tailed arrow). **(B)** The objectives were examined by specified models, including different comparison groups, neurodevelopmental outcomes and regression models. The objectives were motivated both by findings from previous studies and the findings from the preceding aim of the present study. Abbreviations: ADHD: attention-deficit/hyperactivity disorder; ASQ: Ages and Stages Questionnaire; CBCL-DSM: Child Behavior Checklist, Diagnostic and Statistical Manual of Mental Disorders subscale; DNAm: DNA methylation; EWAS: epigenome-wide association study; LCGA: latent class growth analysis; y: years. Created with [BioRender.com](http://www.biorender.com/).


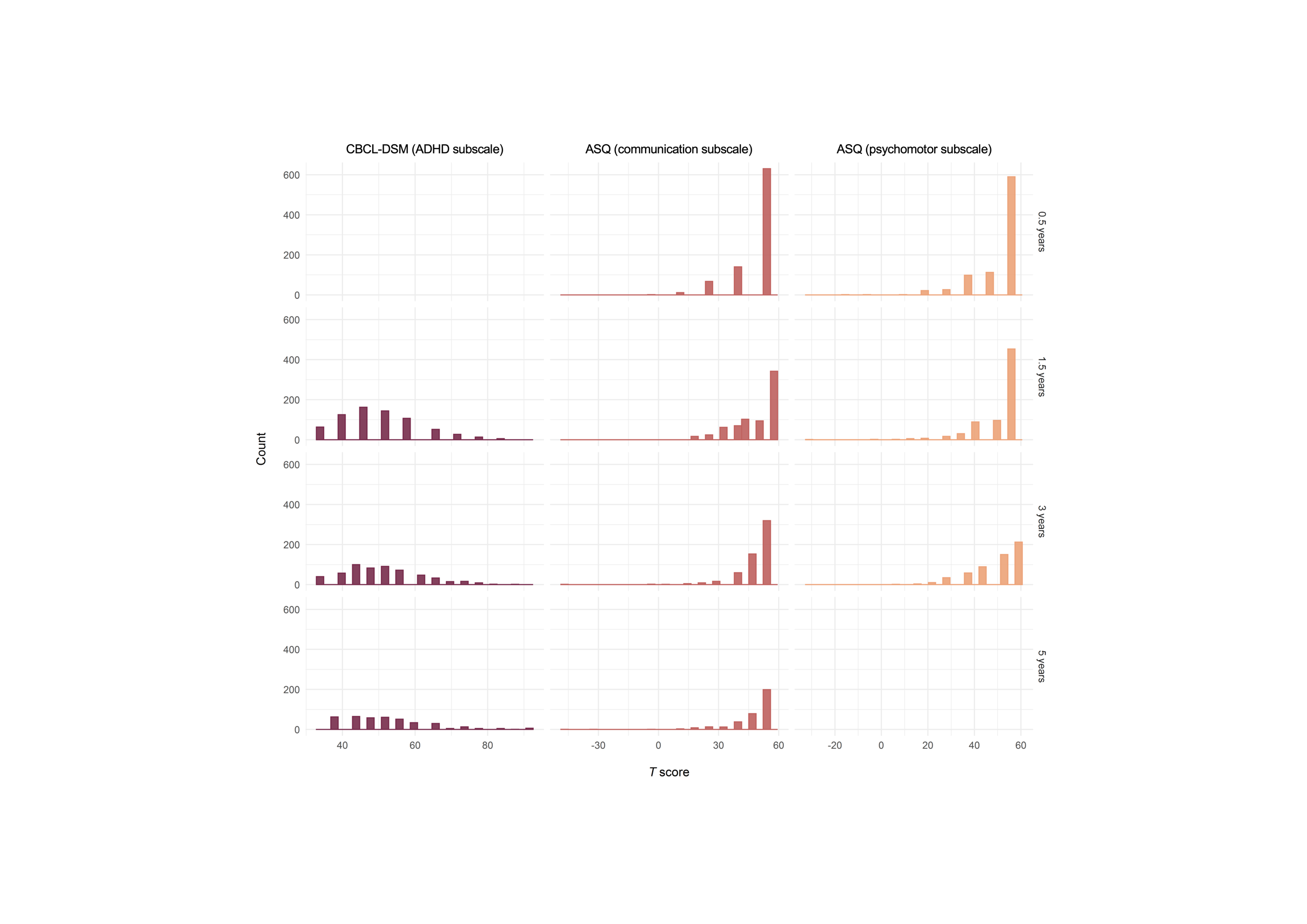


**Supplementary Figure 5. Histograms of T scores for the psychometric tests per timepoint.**

Distribution of T scores for subscales of the CBCL-DSM and ASQ questionnaires (columns) across multiple timepoints (rows). **Abbreviations:** ADHD: attention-deficit/hyperactivity disorder; ASQ: Ages and Stages Questionnaire; CBCL-DSM: Child Behavior Checklist, Diagnostic and Statistical Manual of Mental Disorders subscale.

**
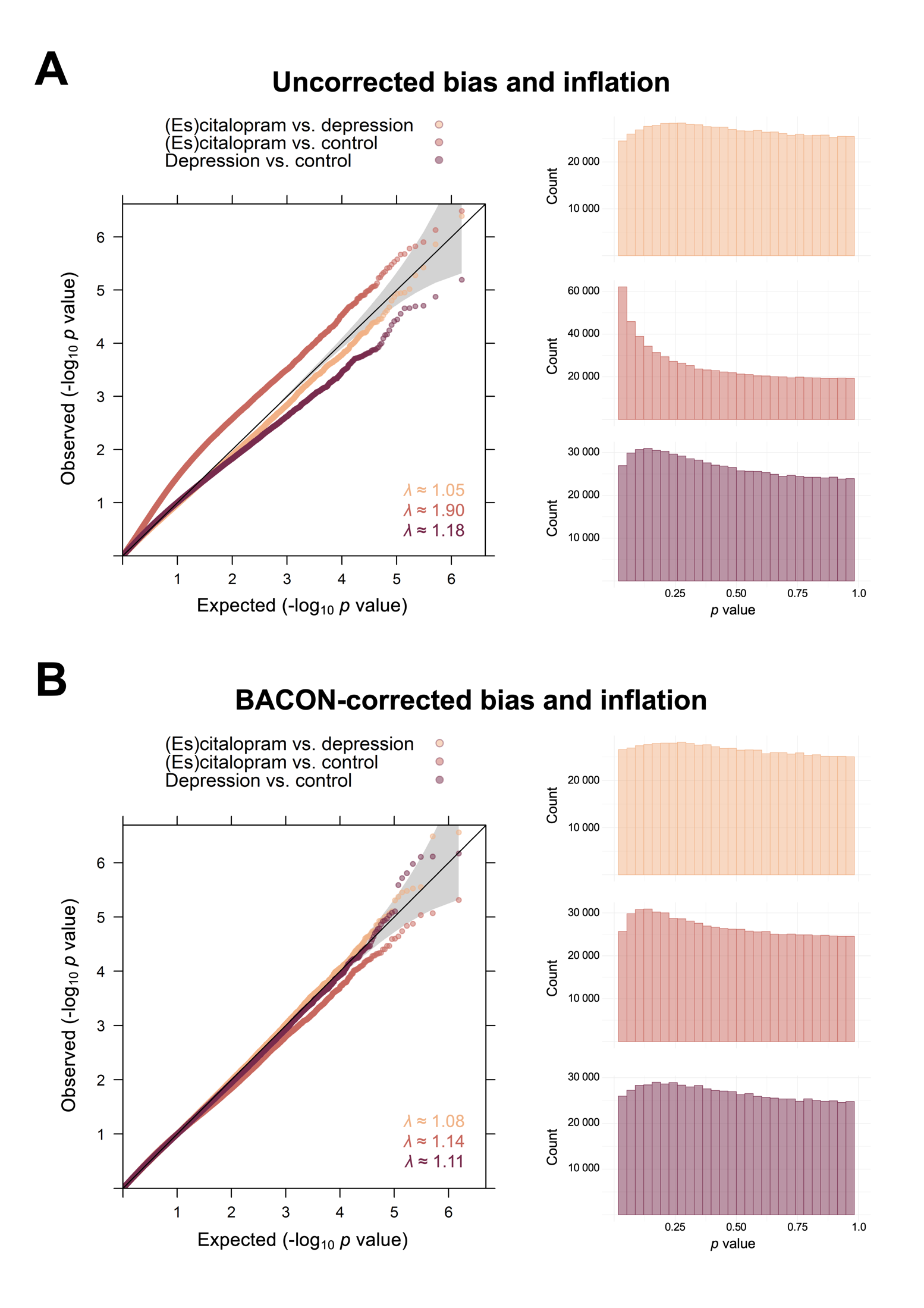
**

**Supplementary Figure 6. QQ plot and *p* value histograms testing DNAm differences between comparison groups.**

Quantile-quantile (QQ) plot and histograms of p values when pairwise testing differences in DNAm between the (es)citalopram (yellow), depression (orange) and control (purple) groups. Plots were generated **(A)** before and **(B)** after correction for bias and inflation using the Bayesian method to correct for test statistic bias and inflation using the R package BACON [14].


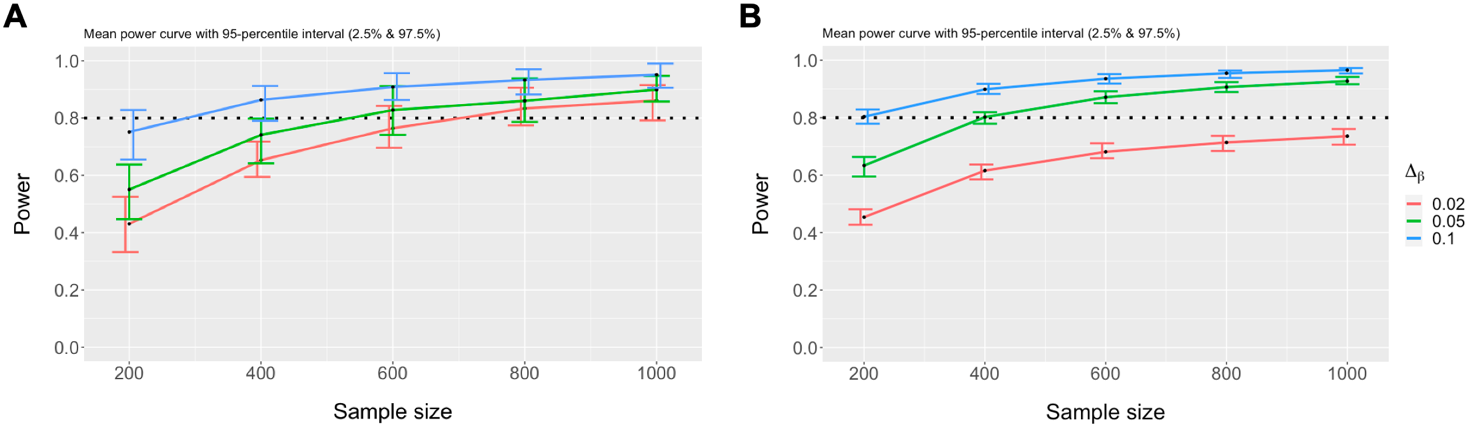


**Supplementary Figure 7. Power calculation to estimate appropriate sample sizes.**

Power calculation for EWASs over increasing total sample sizes (assuming the two comparison groups to have equal sizes), and for various differences in DNAm levels (*Δβ*), when expecting **(A)** 100 significant CpGs and **(B)** 1,000 significant CpGs. Plots were generated using *pwrEWAS* [4].

**Supplementary Tables**

**Supplementary Table 1.** **Overview of the Child Behaviour Checklist (CBCL) items included in the study** [15]**. Only items of the ADHD subscale were included.**

| **Item**  **(Q5/Q6/Q5y)** | **To what extent are the following statements true of your child’s behavior during the last two months?** |
| --- | --- |
| **1 / 2 / 2** | Can’t concentrate, can’t pay attention for long |
| **3 / 3 / 3** | Can’t sit still, restless or overactive |
| **NA / 4 / 4** | Can’t stand waiting, wants everything now |
| **NA / 8 / 8** | Demands must be met immediately |
| **4 / 15 / 14** | Gets into everything |
| **NA / 18 / 24** | Poorly coordinated or clumsy |
| **2 / 20 / 19** | Quickly shifts from one activity to another |

**Response options (score):** “Not true” (0); “Somewhat or sometimes true” (1); “Very true or often true” (2).

**Supplementary Table 2. Overview of the Ages and Stages Questionnaire (ASQ) items included in the study** [7]**. Only items of the communication and psychomotor subscales were included.**

|  | **Item** | **Question** |
| --- | --- | --- |
| **0.5 years** | ***Communication*** | |
|  | **4** | When you “chat” to your child, does he/she try to “chat” back to you? |
|  | **5** | Does your child babble and make sounds when he/she is lying on his/her own? |
|  | **8** | When you call your child, does he/she turn towards you one of the first times you say his/her name? |
|  | ***Gross motor*** | |
|  | **2** | When your child is on his/her tummy, does he/she straighten both arms and push her whole chest off the bed or floor? |
|  | **3** | Does your child roll over from his/her back onto his/her tummy? |
|  | ***Fine motor*** | |
|  | **9** | Does your child grab a toy you offer and then put it in his/her mouth or hold it? |
|  | **10** | When your child is sitting on your lap, does he/she stretch out for a toy or something else on the table in front of you? |
|  | **11** | Does your child hold onto a toy with both hands when he/she is examining it? |
| **1.5 years** | ***Communication*** | |
|  | **1** | When you ask him/her, does your child go into another room to find a familiar toy or object? (You might ask, “Where is your ball?”, or say, “Bring me your coat” or “Go get your blanket”) |
|  | **2** | Does your child say eight or more words in addition to “mama” and “dada”? |
|  | **3** | Without showing him/her first, does your child point to the correct picture when you say, “Show me the kitty” or ask, “Where is the dog”? |
|  | ***Gross motor*** | |
|  | **4** | Does your child move around by walking, rather than by crawling on his/her hands and knees? |
|  | **5** | Can your child walk well and seldom fall? |
|  | **6** | Does your child walk down stairs if you hold onto one of his/her hands? |
|  | ***Fine motor*** | |
|  | **7** | Does your child throw a small ball or toy with a forward arm motion? (If he/she drops the ball, mark “Not yet”) |
|  | **8** | Does your child stack a small block or toy on top of another one? (E.g. small boxes or toys about 3 cm in size) |
|  | **9** | Does your child turn the pages of a book by himself/herself? (He/she may turn more than one page at a time.) |
| **3 years** | ***Communication*** | |
|  | **5** | Without showing him/her first, does your child point to the correct picture when you say, “Where is the cat” or “Where is the dog”? Your child must only point at the correct picture |
|  | **6** | When you ask your child to point to his/her eyes, nose, hair, feet, ears, and so forth, does he/she correctly point to at least seven body parts? (The child can point to parts of himself/herself, you, or a doll.) |
|  | **7** | Does your child make sentences that are three or four words long? |
|  | **8** | Without giving him/her help by pointing or using gestures, ask your child to “Put the shoe on the table” and “Put the book under the chair”. Does your child carry out both of these directions correctly? |
|  | **9** | When looking at a picture book, does your child tell you what is happening or what action is taking place in the picture? (For example, “Barking”, “Running”, “Eating” and “Crying”?) You may ask, “What is the dog (or boy) doing?” |
|  | **10** | Can your child tell you at least two things about an object he/she is familiar with? If you say, for example, “Tell me about your ball”, will your child answer by saying something like “It is round, I can throw it, it is big.” |
|  | ***Gross motor*** | |
|  | **1** | Without holding onto anything for support, does your child kick a ball by swinging his/her leg forward? |
|  | **2** | Can your child catch a large ball with both hands? |
|  | ***Fine motor*** | |
|  | **3** | When drawing, does your child hold a pencil, crayon, or pen between his/her fingers and thumb like an adult does? |
|  | **4** | Can your child undo one or more buttons? |
|  | ***Communication*** | |
| **5 years** | **1** | Can your child tell you at least two things about common object? For example, if you say to your child, “Tell me about the ball”, does he say something like, “It is round. I throw it. It is big”? |
|  | **2** | Without giving your child help by pointing or repeating directions, does your child follow three directions that are unrelated to one another? Give all three directions before your child starts. For example, you may ask your child to “Clap your hands, walk to the door, and sit down” or “Give me the pen, open the book, and stand up.” |
|  | **3** | Does your child use four- and five- word sentences? For example, does your child say, “I want the car”? |
|  | **4** | When talking about something that already happened, does your child use words that end in “ed” such as walked, jumped or played? Ask your child questions, such as “How did you get to the store?” (“We walked.”) “What did you do at your friend’s house?” (“We played.”) |
|  | **5** | Does your child use comparison words, such as heavier, stronger or shorter? Ask your child questions, such as “A car is big, but a bus is _____” (bigger); “A cat is heavy, but a man is ____” (heavier); A TV is small, but a book is ____ ” (smaller). |
|  | **6** | Does your child answer the following questions: 1. “What do you do when you are hungry?” (Acceptable answers include: “Get food”, “Eat”, “Ask for something to eat”, and “Have a snack”.) 2. “What do you do when you are tired?” (Acceptable answers include: “Take a nap”, “Rest”, “Go to sleep”, “Go to bed”, “Lie down”, and “Sit down.”) |
|  | **7** | Does your child repeat the sentences shown below back to you, without any mistakes? You may repeat each sentence one time. Mark “yes” if your child repeats both sentences without mistakes or “sometimes” if your child repeats one sentence without mistakes. “Jane hides her shoes for Maria to find.” “Al read the blue book under his bed.” |

**Response options (score):** “No, not yet” (0); “Yes, but seldom / Sometimes / A few times” (5); “Yes, often” (10).

**Supplementary Table 3. Extended overview of the comparison group characteristics.**

|  | **(Es)citalopram group**  (*n* = 306) | **Depression group**  (*n* = 308) | **Control group**  (*n* = 344) | ***p*** | |
| --- | --- | --- | --- | --- | --- |
| **Maternal characteristics** | | | | | |
| **Maternal age**  (mean years ± SD) | 30.3 ± 5.2 | 28.4 ± 5.3 | 30.9 ± 4.6 | **^a,b^** |  |
| **Pre-pregnancy BMI**  (mean BMI ± SD) | 24.5 ± 5.1  *8 NA* | 24.3 ± 4.8  *7 NA* | 23.8 ± 4.2  *2 NA* | **N.S.** |  |
| **Maternal education**  University/college (*n* (%))  High school or lower (*n* (%)) | 178 (58.2)  124 (40.5)  *4 NA* | 136 (44.2)  163 (52.9)  *9 NA* | 247 (71.8)  89 (25.9)  *8 NA* | **^c,d,e^** |  |
| **Smoking in pregnancy**  (yes; *n* (%)) | 43 (14.1)  *2 NA* | 52 (16.9) | 28 (8.1) | **^f,g^** |  |
| **Alcohol in pregnancy**  (yes; *n* (%)) | 36 (11.8)  *49 NA* | 28 (9.1)  *45 NA* | 64 (18.6)  *15 NA* | **^h^** |  |
| **Folic acid in pregnancy**  (yes; *n* (%)) | 182 (59.5) | 168 (54.6) | 205 (59.6) | **N.S.** |  |
| **Multivitamins in pregnancy**  (yes; *n* (%)) | 109 (35.6) | 127 (41.2) | 119 (34.6) | **N.S.** |  |
| **Maternal medications** | | | | | |
| **Analgesics***  (yes; *n* (%)) | 190 (62.1) | 191 (62.0) | 178 (51.7) | **^I,j^** |  |
| **Antidepressants except (es)citalopram**  (yes; *n* (%)) | 19 (6.2) | --- | --- | **---** |  |
| **NSAIDs**  (yes; *n* (%)) | 55 (18.0) | 45 (14.6) | 30 (8.7) | **^k,l^** |  |
| **Maternal morbidities** | | | | | |
| **Comorbidity index****  (mean score ± SD) | 0.5 ± 0.9  *27 NA* | 0.5 ± 0.9  *6 NA* | 0.4 ± 0.9  *13 NA* | **N.S.** |  |
| **Chronic diseases*****  None (*n* (%))  1–2 diseases (*n* (%))  ≥3 diseases (*n* (%)) | 277 (90.5)  27 (8.8)  0 (0)  *2 NA* | 280 (90.9)  28 (9.1)  0 (0) | 325 (94.5)  19 (5.5)  0 (0) | **N.S.** |  |
| **SCL-5**  (mean score ± SD) | 1.9 ± 0.8  *16 NA* | 2.8 ± 0.5 | 1.0 ± 0 | **^m,n,o^** |  |
| **SCL-8**  (mean score ± SD) | 1.7 ± 0.6  *44 NA* | 2.7 ± 0.5 | 1.0 ± 0 | **^p,q,r^** |  |
| **LTHMD**  (yes; *n* (%)) | 136 (44.4)  *7 NA* | 101 (32.8)  *9 NA* | 114 (33.1)  *1 NA* | **^s,t^** |  |
| **Birth** | | | | | |
| **Caesarean section**  (yes; *n* (%)) | 48 (15.7) | 51 (16.6) | 27 (7.9) | **^u,v^** |  |
| **Child characteristics** | | | | | |
| **Birth weight**  (mean grams ± SD) | 3,568 ± 501 | 3,579 ± 512 | 3,629 ± 503  *1 NA* | **^w^** |  |
| **Gestational age**  (mean weeks ± SD) | 39.4 ± 1.5  *1 NA* | 39.4 ± 1.6  *1 NA* | 39.7 ± 1.5  *1 NA* | **^x,y^** |  |
| **Infant sex**  (female; *n* (%)) | 148 (48.4) | 149 (48.4) | 181 (52.6) | **N.S.** |  |
| **Malformation**  (yes; *n* (%)) | 16 (5.2) | 15 (4,9) | 9 (2.6) | **N.S.** |  |
| **Technical covariates** | | | | | |
| **Bisulphite conversion**  Plate 1 (*n* (%))  Plate 2 (*n* (%))  Plate 3 (*n* (%))  Plate 4 (*n* (%))  Plate 5 (*n* (%))  Plate 6 (*n* (%))  Plate 7 (*n* (%))  Plate 8 (*n* (%))  Plate 9 (*n* (%))  Plate 10 (*n* (%))  Plate 11 (*n* (%))  Plate 12 (*n* (%)) | 30 (9.8)  28 (9.2)  28 (9.2)  3 (1.0)  24 (7.8)  33 (10.8)  29 (9.5)  32 (10.5)  26 (8.5)  13 (4.2)  30 (9.8)  30 (9.8) | 29 (9.4)  32 (10.4)  24 (7.8)  5 (1.6)  29 (9.4)  30 (9.7)  28 (9.1)  32 (10.4)  30 (9.7)  17 (5.5)  27 (8.8)  25 (8.1) | 31 (9.0)  34 (9.9)  38 (11.1)  5 (1.5)  35 (10.2)  28 (8.1)  35 (10.2)  27 (7.9)  30 (8.7)  12 (3.5)  32 (9.3)  37 (10.8) | **N.S.** |  |

**Abbreviations:** ADHD: attention-deficit/hyperactivity disorder; BMI: body mass index; NA: missing value; N.S.: not significant; SD: standard deviation.

* Includes all medications with the N02 Anatomical Therapeutic Chemical (ATC) code except paracetamol (i.e., opioids, antimigraine preparations, and other analgesics and antipyretics)

** Includes all variables available in MBRN and MoBa from a list provided in Bateman *et al.* (2013) [16, 17]. The different variables are given different weights (weight in parentheses).The variables included in the final score are: asthma (1), cardiovascular disease (3), chronic renal disease (1), congenital heart disease (4), illicit substance use (2), gestational hypertension (1), mild-unspecified preeclampsia (2), severe preeclampsia (5), placenta previa (2), pre-existing diabetes mellitus (1), pre-existing hypertension (1), previous Caesarean delivery (1), lupus (2), alcohol abuse (weekly consumption; 1) and maternal age group (> 44 years: 3; 40–45 years: 2; 35–40 years: 1; < 35 years: 0).

*** Chronic diseases included were asthma, rheumatoid arthritis, epilepsy, Crohn’s disease, lupus, multiple schlerosis (MS), cancer and diabetes mellitus. All diseases were weighted equally and each additional disease added 1 to the final score.

^a^ *p* < 0.0001, comparing (es)citalopram to depression

^b^ *p* < 0.0001, comparing depression to controls

^c^ *p* ≈ 0.001, comparing (es)citalopram to depression

^d^ *p* < 0.0001, comparing (es)citalopram to controls

^e^ *p* < 0.0001, comparing depression to controls

^f^ *p* ≈ 0.02, comparing (es)citalopram to controls

^g^ *p* ≈ 0.001, comparing depression to controls

^h^ *p* ≈ 0.01, comparing depression to controls

^i^ *p* ≈ 0.01, comparing (es)citalopram to controls

^j^ *p* ≈ 0.01, comparing depression to controls

^k^ *p* ≈ 0.001, comparing (es)citalopram to controls

^l^ *p* ≈ 0.03, comparing depression to controls

^m^ *p* < 0.0001, comparing (es)citalopram to controls

^n^ *p* < 0.0001, comparing (es)citalopram to controls

^o^ *p* < 0.0001, comparing depression to controls

^p^ *p* < 0.0001, comparing (es)citalopram to depression

^q^ *p* < 0.0001, comparing (es)citalopram to controls

^r^ *p* < 0.0001, comparing depression to controls

^s^ *p* ≈ 0.004, comparing (es)citalopram to depression

^t^ *p* ≈ 0.002, comparing (es)citalopram to controls

^u^ *p* ≈ 0.003, comparing (es)citalopram to controls

^v^ *p* ≈ 0.001, comparing depression to controls

^w^ *p* ≈ 0.05, comparing (es)citalopram to controls

^x^ *p* ≈ 0.001, comparing (es)citalopram to controls

^y^ *p* ≈ 0.03, comparing depression to controls

**Supplementary Table 4.** **Overview of the cell type proportions of the comparison groups.**

|  | **(Es)citalopram**  (*n* = 306) | **Depression control**  (*n* = 308) | **Healthy control**  (*n* = 344) | ***p*** |
| --- | --- | --- | --- | --- |
| **B cells;**  *mean proportion ± SD* | 0.04 ± 0.02 | 0.04 ± 0.02 | 0.04 ± 0.02 | **N.S.** |
| **CD4^+^ T cells;**  *mean proportion ± SD* | 0.12 ± 0.05 | 0.13 ± 0.05 | 0.13 ± 0.05 | **N.S.** |
| **CD8^+^ T cells;**  *mean proportion ± SD* | 0.05 ± 0.02 | 0.05 ± 0.03 | 0.05 ± 0.02 | **N.S.** |
| **Granulocytes;**  *mean proportion ± SD* | 0.57 ± 0.10 | 0.57 ± 0.10 | 0.58 ± 0.10 | **N.S.** |
| **Monocytes;**  *mean proportion ± SD* | 0.06 ± 0.03 | 0.06 ± 0.03 | 0.06 ± 0.03 | **N.S.** |
| **Natural killer cells;** *mean proportion ± SD* | 0.02 ± 0.02 | 0.03 ± 0.03 | 0.03 ± 0.02 | **N.S.** |
| **nRBCs;**  *mean proportion ± SD* | 0.12 ± 0.11 | 0.11 ± 0.10 | 0.10 ± 0.10 | **^a,b^** |

**Abbreviations:** ADHD: attention-deficit hyperactivity disorder; nRBC: nucleated red blood cell; N.S.: not significant; SD: standard deviation.

^a^ *p* < 0.01, (es)citalopram to controls

^b^ *p* < 0.05, depression to controls

**Supplementary Table 5. Model specification and fit, classification quality and distribution across classes for the latent class growth analysis of the CBCL-DSM ADHD subscale (*n* = 786). The selected model is shaded in grey.**

| **Model specifications** | | **Goodness-of-fit/classification quality** | | | | **Classification (% of samples)** | | | | |
| --- | --- | --- | --- | --- | --- | --- | --- | --- | --- | --- |
| **Number of classes** | **Time**  **function** | **AIC** | **BIC** | **c-BIC** | **Entropy** | **Class 1** | **Class 2** | **Class 3** | **Class 4** | **Class 5** |
| **1** | Linear | 9430.7 | 9547.4 | 9468.0 | 1.00 | 100.0 | --- | --- | --- | --- |
| **1** | 2^nd^ degree polynomial | 9432.5 | 9553.8 | 9471.2 | 1.00 | 100.0 | --- | --- | --- | --- |
| **2** | Linear | 9243.3 | 9373.9 | 9285.0 | 0.52 | 63.0 | 37.0 | --- | --- | --- |
| **2** | 2^nd^ degree polynomial | 9247.2 | 9387.2 | 9291.9 | 0.52 | 63.1 | 36.9 | --- | --- | --- |
| **3** | Linear | 9193.5 | 9338.2 | 9239.8 | 0.59 | 66.9 | 10.6 | 22.5 | --- | --- |
| **3** | 2^nd^ degree polynomial | 9199.2 | 9357.8 | 9249.9 | 0.59 | 69.7 | 19.6 | 10.7 | --- | --- |
| **4** | Linear | 9186.3 | 9344.9 | 9237.0 | 0.58 | 3.7 | 23.8 | 63.5 | 9.0 | --- |
| **4** | **2^nd^ degree polynomial** | **9170.1** | **9347.5** | **9226.8** | **0.69** | **9.5** | **7.9** | **71.2** | **11.3** | **---** |
| **5** | Linear | 9178.0 | 9350.7 | 9233.2 | 0.64 | 40.2 | 0.5 | 3.7 | 48.5 | 7.1 |
| **5** | 2^nd^ degree polynomial | *Did not converge* | | | | --- | --- | --- | --- | --- |

**Abbreviations:** AIC: Akaike information criterion; BIC: Bayesian information criterion; c-BIC: sample size-corrected Bayesian information criterion; LCGA: Latent class growth analysis.

**Supplementary Table 6. Model specification and fit, classification quality and distribution across classes for the latent class growth analysis of the ASQ communication subscale (*n* = 899). The selected model is shaded in grey.**

| **Model specifications** | | **Goodness-of-fit/classification quality** | | | | **Classification (% of samples)** | | | | |
| --- | --- | --- | --- | --- | --- | --- | --- | --- | --- | --- |
| **Number of classes** | **Time**  **function** | **AIC** | **BIC** | **c-BIC** | **Entropy** | **Class 1** | **Class 2** | **Class 3** | **Class 4** | **Class 5** |
| **1** | Linear | 9910.6 | 10030.6 | 9951.2 | 1.00 | 100.0 | --- | --- | --- | --- |
| **1** | 2^nd^ degree polynomial | 9904.1 | 10028.9 | 9946.3 | 1.00 | 100.0 | --- | --- | --- | --- |
| **2** | Linear | 9784.6 | 9919.1 | 9830.2 | 0.49 | 27.6 | 72.4 | --- | --- | --- |
| **2** | 2^nd^ degree polynomial | 9690.4 | 9834.4 | 9739.1 | 0.54 | 55.2 | 44.8 | --- | --- | --- |
| **3** | **Linear** | **9765.2** | **9914.1** | **9815.6** | **0.66** | **28.8** | **70.5** | **0.7** | **---** | **---** |
| **3** | 2^nd^ degree polynomial | 9669.8 | 9833.0 | 9725.0 | 0.68 | 1.0 | 43.8 | 55.2 | --- | --- |
| **4** | Linear | 9771.2 | 9934.5 | 9826.5 | 0.68 | 28.8 | 70.5 | 0.7 | 0.0 | --- |
| **4** | 2^nd^ degree polynomial | 9677.8 | 9860.2 | 9739.5 | 0.55 | 44.9 | 1.0 | 54.1 | 0.0 | --- |
| **5** | Linear | 9777.2 | 9954.9 | 9837.4 | 0.72 | 28.8 | 0.0 | 70.5 | 0.0 | 0.7 |
| **5** | 2^nd^ degree polynomial | 9685.8 | 9887.4 | 9754.0 | 0.44 | 61.6 | 1.0 | 0.0 | 37.4 | 0.0 |

**Abbreviations:** AIC: Akaike information criterion; BIC: Bayesian information criterion; c-BIC: sample size-corrected Bayesian information criterion; LCGA: Latent class growth analysis.

**Supplementary Table 7. Model specification and fit, classification quality and distribution across classes for the latent class growth analysis of the ASQ motor subscale (*n* = 895). The selected model is shaded in grey.**

| **Model specifications** | | **Goodness-of-fit/classification quality** | | | | **Classification (% of samples)** | | | | |
| --- | --- | --- | --- | --- | --- | --- | --- | --- | --- | --- |
| **Number of classes** | **Time**  **function** | **AIC** | **BIC** | **c-BIC** | **Entropy** | **Class 1** | **Class 2** | **Class 3** | **Class 4** | **Class 5** |
| **1** | Linear | 8091.7 | 8202.0 | 8129.0 | 1.00 | 100.0 | --- | --- | --- | --- |
| **1** | 2^nd^ degree polynomial | 8085.9 | 8201.0 | 8124.8 | 1.00 | 100.0 | --- | --- | --- | --- |
| **2** | Linear | 7492.2 | 7617.0 | 7534.4 | 0.69 | 27.9 | 72.1 | --- | --- | --- |
| **2** | 2^nd^ degree polynomial | 7177.5 | 7311.8 | 7222.8 | 0.66 | 65.5 | 34.5 | --- | --- | --- |
| **3** | Linear | 7383.6 | 7522.7 | 7430.6 | 0.66 | 15.8 | 30.1 | 54.2 | --- | --- |
| **3** | 2^nd^ degree polynomial | 7050.8 | 7204.3 | 7102.7 | 0.63 | 16.9 | 53.1 | 30.1 | --- | --- |
| **4** | Linear | 7374.1 | 7527.6 | 7426.0 | 0.65 | 18.0 | 54.0 | 24.4 | 3.7 | --- |
| **4** | 2^nd^ degree polynomial | 7014.1 | 7186.8 | 7072.4 | 0.67 | 15.6 | 47.7 | 7.9 | 28.7 | --- |
| **5** | Linear | 7365.2 | 7533.1 | 7421.9 | 0.70 | 0.1 | 53.9 | 18.0 | 25.1 | 2.9 |
| **5** | **2^nd^ degree polynomial** | **6966.2** | **7158.1** | **7031.1** | **0.68** | **15.1** | **8.2** | **50.2** | **12.3** | **14.3** |

**Abbreviations:** AIC: Akaike information criterion; BIC: Bayesian information criterion; c-BIC: sample size-corrected Bayesian information criterion; LCGA: Latent class growth analysis.

**Supplementary Table 8. Listed significant CpGs from the models run in the study (statistics and annotation).**

*Please see separate .xlsx file (“Supplementary Information, Supplementary Table 8.xlsx”).*

**Supplementary Table 9. Distribution of comparison groups in the trajectory classes identified for the CBCL-DSM ADHD subscale using latent class growth analysis.**

|  | **Class 1**  (*n* = 75) | **Class 2**  (*n* = 62) | **Class 3**  (*n* = 560) | **Class 4**  (*n* = 89) | ***p*** |
| --- | --- | --- | --- | --- | --- |
| **Group**  (Es)citalopram (*n* (%))  Depression (*n* (%))  Control (*n* (%)) | 21 (28.0)  18 (24.0)  36 (48.0) | 24 (38.7)  9 (14.5)  29 (46.8) | 172 (30.7)  162 (28.9)  226 (40.4) | 23 (25.8)  44 (49.4)  22 (24.7) | **N.S.**  **<0.001**  **<0.01** |
| **ADHD diagnosis**  (yes; *n* (%)) | 0 (0) | 2 (3.2) | 29 (5.2) | 12 (13.5) | **<0.001** |

*n* = 786, as women not answering to the CBCL at any of the time points were removed from the analysis.

**Abbreviations:** ADHD: attention-deficit/hyperactivity disorder; N.S.: not significant.

**Supplementary Table 10. Distribution of comparison groups in the trajectory classes identified for the ASQ communication subscale using latent class growth analysis.**

|  | **Class 1**  (*n* = 259) | **Class 2**  (*n* = 634) | **Class 3**  (*n* = 6) | ***p*** |
| --- | --- | --- | --- | --- |
| **Group**  (Es)citalopram (*n* (%))  Depression (*n* (%))  Control (*n* (%)) | 87 (33.6)  84 (32.4)  88 (34.0) | 188 (29.7)  191 (30.1)  255 (40.2) | 1 (16.7)  4 (66.7)  1 (16.7) | **N.S.**  **N.S.**  **N.S.** |

*n* = 899; women not answering to the ASQ at any of the time points were removed from the analysis.

**Abbreviations:** N.S.: not significant.

**Supplementary Table 11. Distribution of comparison groups in the trajectory classes identified for the ASQ total motor subscale using latent class growth analysis.**

|  | **Class 1**  (*n* = 135) | **Class 2**  (*n* = 73) | **Class 3**  (*n* = 449) | **Class 4**  (*n* = 110) | **Class 5**  (*n* = 128) | ***p*** |
| --- | --- | --- | --- | --- | --- | --- |
| **Group**  (Es)citalopram (*n* (%))  Depression (*n* (%))  Control (*n* (%)) | 45 (33.3)  49 (36.3)  41 (30.4) | 26 (35.6)  23 (31.5)  24 (32.9) | 129 (28.7)  145 (32.3)  175 (39.0) | 35 (31.8)  29 (26.4)  46 (41.8) | 40 (31.3)  32 (25.0)  56 (43.8) | **N.S.**  **N.S.**  **N.S.** |

*n* = 895; women not answering to the ASQ at any of the time points were removed from the analysis.

**Abbreviations:** N.S.: not significant.

**Supplementary Table 12.** **BECon output: blood-brain correlation of the significant CpGs identified in the study.**

*Please see separate .xlsx file (“Supplementary Information, Supplementary Table 12.xlsx”).*
